# Dynamic nanomechanical characterization of cells in exosome therapy

**DOI:** 10.1038/s41378-024-00735-z

**Published:** 2024-07-15

**Authors:** Ye Chen, Zihan Zhang, Ziwei Li, Wenjie Wu, Shihai Lan, Tianhao Yan, Kainan Mei, Zihan Qiao, Chen Wang, Chuanbiao Bai, Ziyan Li, Shangquan Wu, Jianye Wang, Qingchuan Zhang

**Affiliations:** 1 CAS Key Laboratory of Mechanical Behavior and Design of Material, Department of Modern Mechanics, University of Science and Technology of China, Hefei, Anhui 230027 China; 2 Reproductive Medicine Center, Department of Obstetrics and Gynecology, The First Affiliated Hospital of Anhui Medical University, Hefei, 230022 China; 3 Anhui Province Key Laboratory of Reproductive Health and Genetics, Anhui Medical University, Hefei, 230022 China; 4 Department of Cell Biology and Genetics, College of Basic Medical Sciences, Jilin University, Changchun, 130021 China; 5 State Key Laboratory of Nonlinear Mechanics, Institute of Mechanics, Chinese Academy of Science, 15 Beisihuan West Road, Beijing, 100190 China

**Keywords:** Nanoparticles, Optical physics

## Abstract

Exosomes derived from mesenchymal stem cells (MSCs) have been confirmed to enhance cell proliferation and improve tissue repair. Exosomes release their contents into the cytoplasmic solution of the recipient cell to mediate cell expression, which is the main pathway through which exosomes exert therapeutic effects. The corresponding process of exosome internalization mainly occurs in the early stage of treatment. However, the therapeutic effect of exosomes in the early stage remains to be further studied. We report that the three-dimensional cell traction force can intuitively reflect the ability of exosomes to enhance the cytoskeleton and cell contractility of recipient cells, serving as an effective method to characterize the therapeutic effect of exosomes. Compared with traditional biochemical methods, we can visualize the early therapeutic effect of exosomes in real time without damage by quantifying the cell traction force. Through quantitative analysis of traction forces, we found that endometrial stromal cells exhibit short-term cell roundness accompanied by greater traction force during the early stage of exosome therapy. Further experiments revealed that exosomes enhance the traction force and cytoskeleton by regulating the Rac1/RhoA signaling pathway, thereby promoting cell proliferation. This work provides an effective method for rapidly quantifying the therapeutic effects of exosomes and studying the underlying mechanisms involved.

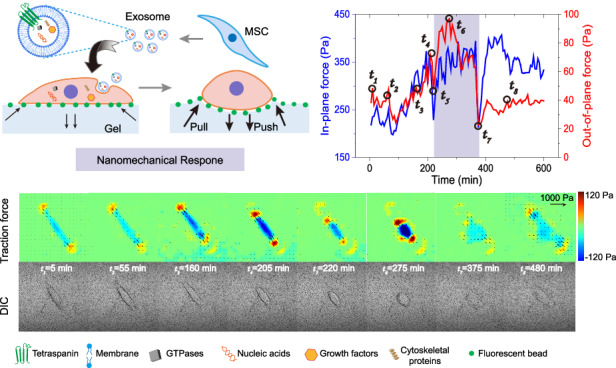

## Introduction

Stem cell therapy, an emerging treatment, is a promising method for treating central nervous system diseases and cardiovascular diseases and repairing damaged tissue; however, effective treatments for these conditions are lacking^1–3^. Previous research has suggested that stem cells can differentiate and replace damaged tissue. However, further studies have shown that exosomes derived from MSCs play an essential role in stem cell therapy^4,5^. Exosomes are extracellular vesicles with a diameter of ~100 nm that contain nucleic acids, proteins, lipids, and metabolites and play an important role in regulating intercellular communication^6,7^. In recent years, elucidation of the mechanism by which exosomes regulate the biochemical pathway of recipient cells has further enhanced the potential of using exosomes for treatment and diagnosis, which is highly important for curing diseases^8,9^.

MSCs can proliferate and differentiate into stem cells and can be isolated from a variety of tissues, making mesenchymal stem cell therapy widely used in regenerative medicine^10^. Currently, animal experiments have shown that abdominal transplantation of MSCs can promote the repair of endometrial damage and increase the pregnancy rate^11–13^, while exosomes secreted by MSCs have also been confirmed to promote wound healing and reduce the inflammatory response^14,15^. Our previous studies have shown that exosomes derived from human umbilical cord mesenchymal stem cells (hUCMSC-Exos) play a positive role in the in vitro repair of endometrial stromal cells (EndoSCs), but their molecular mechanism remains to be further explored^16^. Due to the heterogeneity of exosome origins, the evaluation of exosome therapeutic effects is very important and can assist in the study of the active components of exosomes and ensure the effects of exosomes^17,18^. At the same time, the therapeutic effect of exosomes primarily depends on the direct release of nucleic acids, proteins, lipids, and metabolites into the cytoplasmic solution of the recipient cells to mediate the corresponding biological responses, which often occur in the early stages of action^19^. The development of high-sensitivity methods to analyze the early therapeutic effect of exosome therapy will accelerate the pace of exosome research and clinical applications. Currently, methods including cell viability assays, analysis of protein and gene expression, histological examination and animal experiments have been used to study the therapeutic effectiveness of exosomes, confirming the potential of exosome therapy. However, these methods have certain limitations, including long experimental cycles and challenges in dynamic characterization of the early interactions between exosomes and cells. For example, cell viability detection methods such as MTT and CCK-8 assays require time for the incubation of reagents with cells, and metabolic activity responses to exosomes also take time, making it difficult to effectively characterize early changes in cell activity. Western blotting involves cell lysis and cannot provide dynamic observations of cell status. Animal experiments are time-consuming, demanding, and require a certain period of time for the exosomes to exert reparative effects at the tissue level, making it challenging to assess early exosome effects. Therefore, to further study the mechanism of exosome therapy, it is necessary to develop a dynamic and nondestructive method to precisely evaluate the effect of exosomes.

In the process of life, cells not only have biochemical interactions with the outside world but also have mechanical interactions with the outside matrix. When the structure and function of cells change, the interaction between cells and the outer matrix will also change^20,21^. By analyzing the interaction between cells and the outside matrix, the physiological state of cells can be determined. In fact, in our team’s early studies based on a microcantilever sensor, it was found that the micromotion of cancer cells and sperm could cause mechanical vibration of the microcantilever, and such vibration was significantly inhibited after anticancer drugs were applied to cancer cells^22,23^. Wu et al. also showed that in the early stage of chemotherapy, the cell traction force can be used as an effective indicator of the effect of chemotherapy drugs^24^. Our team developed a method that utilizes fluorescent particles in a single layer to quantify the three-dimensional traction forces generated by cancer cells during mitosis^25–27^. Using this method, it was found that cell traction forces exhibit distinct changes in response to variations in cytoskeleton structure. This finding further indicates that the mechanical interaction between cells and the extracellular microenvironment is directly influenced by cell structure. Previous studies have shown that exosome treatment of stem cells can promote microtubule polymerization and increase cell activity^28–30^, which is likely to have a positive effect on the mechanical interaction between cells and the outside world. The effective acquisition of these mechanical signals is expected to evaluate the effect of exosome therapy and reflect the characteristic changes in the internal structure of cells. Therefore, we investigated the characterization of the therapeutic effect of exosomes through the mechanical interaction between cells and the extracellular matrix to assist in the study of the early therapeutic mechanism of exosomes.

Here, we quantified the three-dimensional traction force of EndoSCs to characterize the therapeutic effect of exosomes derived from MSCs. In combination with traditional methods of measuring cell activity and proliferation, we investigated the response of cellular mechanical and biological signals when treated with exosomes for different periods of time (2 h and 24 h). On this basis, we observed a transient cell rounding phenomenon in the early stages of exosome treatment of injured cells. We further investigated the relationship between cell rounding and cell traction force and explored the impact of specific proteins by fluorescence staining. Moreover, we studied the activation of Rac1 and RhoA after treatment with exosomes by pull-down and western blot experiments and explored their role in cell proliferation.

## Materials and methods

### Materials and reagents

3-Aminopropyltrimethoxysilane (APTES), sodium dodecyl sulfate (SDS) and N-hydroxysuccinimide (NHS) were purchased from Sigma‒Aldrich (St. Louis, MO, USA). Glass-bottom culture dishes were obtained from Nest Biotechnology Co., Ltd. (Wuxi, China). Phosphate-buffered saline (PBS; 0.1 M phosphate buffer containing 0.9% sodium chloride, pH 7.5) and N-(3-dimethylaminopropyl)-N’-ethylcarbodiimide hydrochloride (EDC) were obtained from Sangon Biotech Co., Ltd. (Shanghai, China). Rattail tendon collagen type I was purchased from Shanghai Canspec Scientific Instruments Co., Ltd. (Shanghai, China). Sulfosuccinimidyl 6-(4-azido-2-nitrophenyl-amino) hexanoate, Hoechst 33258, CellMask™ Deep Red Actin Tracking Stain, and carboxylate-modified green fluorescent beads (0.49 μm in diameter, 505/515 nm, F-8813 molecular probes) were obtained from Thermo Fisher Scientific (Waltham, MA, USA). SiR-Tubulin, SiR-Actin, Rac1 Activation Assay Biochem Kit™, and Rho Activation Assay Biochem Kit™ were purchased from Cytoskeleton (Denver, CO, USA). Anti-rabbit IgG (H + L) F(ab’)2 fragment (Alexa Fluor® 488 Conjugate) #4412 and anti-mouse IgG (H + L) F(ab’)2 fragment (Alexa Fluor® 647 Conjugate) #4410 were purchased from Cell Signaling Technology (Beverly, MA, USA). Rabbit monoclonal anti-paxillin (1/200 dilution) (Ab32084), rabbit monoclonal anti-nonmuscle myosin IIA (1/200 dilution) (Ab138498), and mouse monoclonal anti-F-actin (1/200 dilution) (Ab205) were purchased from Abcam (Cambridge, UK). NSC23766 (Cat# HY-15723), Y27632 (Cat# HY-10071), and calyculin A (Cat# HY-18983) were purchased from MedChemExpress. All other chemicals were obtained from Beijing Chemical Reagents Co., Ltd. (Beijing, China).

### Preparation of the substrate

A glass-bottom culture dish was pretreated with 2 mL of 4% (v/v) APTES solution in deionized water for 15 min. The Petri dish was washed 3 times with deionized water before it was covered with 2 mL of 0.5% glutaraldehyde solution in PBS for 30 min. The Petri dish was subsequently washed with deionized water and allowed to dry naturally.

The polyacrylamide (PAA) hydrogel was prepared according to previous protocols^31,32^. A solution of 5% (w/v) acrylamide, 0.5% (w/v) *N*,*N*-methylene-bis-acylamide, 0.5% *N*,*N*,*N*’,*N*’-tetramethylethylenediamine, and 0.05% ammonium persulfate was mixed to form the pregel solution. The pregel solution was carefully placed between a fixed glass plate and a Petri dish and then polymerized for 60 min at room temperature. The PAA gel was ~80–100 μm thick and was tested by an atomic force microscope (Bruke, USA). The Young’s modulus of the PAA gel was ~5.8 kPa, which was tested by a rheometer (MCR 302, Anton Paar, Austria). Next, 4 μL of green fluorescent beads were fully diluted with 200 μL of deionized water and modified on the surface of the PAA gels for 60 min. To activate the carboxylic acid groups, a mixture of 0.5% NHS and 2% EDC in water was added to the surface of the PAA gels for 3 h in the dark. To promote cell adhesion on the PAA gels, a 1 mg/mL solution of sulfo-SANPAH in water (200 μL) was pipetted onto the PAA gel surface under ultraviolet (UV) light (10 W) for 10 min. Then, the Petri dish was washed 3 times with deionized water, and a solution of 0.6% (w/v) collagen I in 200 μL of aqueous acetic acid (6 mM) was incubated on the gels overnight at 4 °C.

### Cell culture

Healthy endometrial tissue at the proliferative stage was cut into small pieces and incubated for 1 h with 0.1% type I collagenase (Sigma, USA). Then, dispersed EndoSCs were separated by filtration with a screen (180 μm and 40 μm). DMEM/F-12 cell medium containing 10% fetal bovine serum (Gibco, USA) and 1% penicillin/streptomycin (HyClone, USA) was prepared for cell culture. Cell-specific markers, such as CD34, CD44, and CD90, were identified by flow cytometry (BD FACSCalibur, USA).

The EndoSCs were cultured to the 3rd–5th generation. When the cells grew well, they were transferred to gel culture dishes. When the cell confluence reached ~80%, the media in the gel culture dishes were replaced with DMEM/F12 medium supplemented with 60 μmol/L mifepristone (Sigma, USA) for 48 h. The endometrial stromal cell injury model was established in vitro.

Transformed human corneal epithelial cells (HCE-T, Cellcook cat: CC4018) were cultured in DMEM/F12 (Gibco cat: 11330) supplemented with 10% fetal bovine serum, 10 ng/mL epidermal growth factor (EGF, MCE), and 5 µg/mL insulin (MCE) according to the manufacturer’s recommendations. MCF-10A cells (ATCC) were cultured with complete MCF-10A medium (Cellcook). The cell culture procedure used before direct exosome treatment was similar to that used for the above injury model, but the mifepristone was removed.

### Time-lapse image acquisition

All confocal images for calculating the cell traction force were collected with a Zeiss 40× objective (Plan-Apochromat 40×/0.95 Korr M27) in an inverted Zeiss LSM 800 confocal microscope equipped with a Zeiss CCD camera. The confocal Petri dish was attached to an accompanying live cell workstation (Tokai Hit, Shizuoka, Japan) at 37 °C with 5% CO_2_. An argon laser (488 nm) was used to image the green fluorescent beads. 3D image stacks were acquired every 5 min at a resolution of 512 × 512 × 8 voxels, which corresponds to voxel dimensions of 0.4 μm × 0.4 μm × 0.3 μm in both the horizontal and axial planes. After the acquisition of the time-lapse images, SDS was used to lyse the cells, and images of the undeformed cells were collected. Differential interference contrast (DIC) images or F-actin living cell staining images were recorded to indicate the location and shape of the cells. The confocal images for immunofluorescence analysis were collected on a Leica TCS SP8 laser scanning confocal microscope (Leica Microsystems, Mannheim, Germany). The images were recorded by using a 63×/1.40 NA oil immersion objective lens with a resolution of 1024 × 1024 pixels. An argon laser (405/488/638 nm) was used to excite the antibodies.

### Displacement, stress, and force calculations

All the stress and force results were calculated from displacements, so we referred to the digital volume correlation (DVC) methods in several works to obtain accurate displacements^26,27,33,34^. Based on the displacements, the full-field stress results were obtained by 2.5D Fourier transform traction cytometry methods^35^. The area of cell traction stress was determined by DIC images or F-actin staining images. All displacement results were calculated in MATLAB, and the stress results were calculated in Python. The overall cell traction force was calculated by the stress in the cell region, ${\bar{F}}_{{xy}}=\frac{\iint \sqrt{{F}_{x}^{2}\left(x,y\right)+{F}_{y}^{2}\left(x,y\right)}{dxdy}}{S}$, ${\bar{F}}_{z}=\frac{\iint \sqrt{{F}_{z}^{2}\left(x,y\right)}{dxdy}}{S}$, where ${\bar{F}}_{{xy}}$ is the in-plane force of the cell and ${\bar{F}}_{z}$ is the out-of-plane force of the cell. *S* represents the size of the cell area. ${F}_{x}$, ${F}_{y}$ and ${F}_{z}$ denote the stress results in the cell region.

### Exosome isolation and purification

Human umbilical cords were collected from patients who provided informed consent and were reviewed and approved by the Ethics Committee of Anhui Medical University. The isolation and characterization of hUCMSCs were performed as previously described^16^. Warton’s jelly was separated from the umbilical cord and cut into small pieces. Umbilical cord segments were adhered to cell culture plates (Corning, USA) and cultured for several days in Dulbecco’s modified Eagle’s medium (DMEM/F-12) supplemented with 10% fetal bovine serum (FBS; Gibco, USA). hUCMSCs migrated from Wharton’s jelly blocks. hUCMSCs are characterized by the expression of typical markers and multilineage differentiation potential (adipogenic, osteogenic, and chondrogenic differentiation). hUCMSC-derived exosomes were isolated from exosome-free media using an exosome extraction and purification kit (Umibio, Shanghai, China). The exosomes were characterized by negative-stain electron microscopy and western blotting based on their size and surface marker expression. The exosomes were placed into a formvar/carbon-coated grid, negatively stained with 3% aqueous phosphor-tungstic acid for 1 min, and examined by transmission electron microscopy (TEM, FEI, USA) at an accelerating voltage of 120 kV. The particle size distribution of the hUCMSC-Exos was calculated by nanoparticle tracking analysis (NTA, Zeta View PMX 110, Germany). Typical exosomal markers, such as CD63 (1:1000, Abcam, UK) and TSG101 (1:1000, Abcam, UK), were detected by western blotting. The initial concentration of the exosomes was 12 mg/mL.

For labeling, the exosome solution was incubated with 5 µg/µl Dil (Bestbio, Shanghai, China) for 30 min on ice. The unincorporated dyes were removed using 100-kDa ultrafiltration tubes (Amicon® Ultra) and washed in PBS via ultracentrifugation. The concentrated solutions were diluted in PBS.

### Western blotting

After the proteins were added to the cells, the cells were collected into EP tubes, and the protein concentration was determined by the BCA method. Proteins were separated by 12.5% SDS‒PAGE and transferred to PVDF membranes. The membrane was washed in TBST and blocked with 5% nonfat dry milk in TBST for 120 min at room temperature. Then, the cells were incubated with antibodies against RAC1 (1:500, Cytoskeleton, USA), RhoA (1:500, Cytoskeleton, USA), tubulin (1:1000, Cell Signaling, UK), β-actin (1:1000, Cell Signaling, USA) and GAPDH (1:2000, Cell Signaling, USA) at 4 °C overnight. After being washed 3 times in TBST with shaking, the membrane was incubated with horseradish peroxidase (HRP)-conjugated anti-rabbit/mouse antibodies for 1 h. The membrane was washed again, and the proteins were imaged with an enhanced chemiluminescence detection kit (Biosharp, USA).

### Pull-down assays

A RhoA/Rac1 Activation Assay Biochem Kit (Cytoskeleton, Denver, CO, USA) was used following the manufacturer’s instructions. Briefly, cells were added to cell lysates, scraped, and centrifuged, and rhotekin-RBD (for RhoA activation) or PAK-PBD beads (for Rac1 activation) were added to the lysates. The mixture was incubated at 4 °C on a rotator for 1 h. Then, the beads were centrifuged and washed with PBS. The bead pellets were resuspended in 20 μL of 2× Laemmli sample buffer and boiled. The samples were analyzed by western blot analysis.

### Cell counting Kit-8 (CCK-8) assay

Cell proliferation was measured by a CCK-8 assay (Biomiky, Hefei, China). In brief, 3000 cells/well (5 replicates per group) were inoculated into 96-well plates and established using mifepristone. The group with no cells was used as a blank. After different treatments, CCK-8 solution (10 μL) and 100 μL of DMEM/F12 were added to each well, and the plates were incubated at 37 °C for 2 h. Cell proliferation was determined by subtracting the optical density of the blank well from the absorbance of the test well. A microplate reader (SpectraMAX ID3, USA) was used to measure the optical density (OD) at 450 nm.

### Flow cytometry (FCM) analysis

An Annexin V-FITC/PI apoptosis detection kit (Keygen Biotechnology, China) was used to detect cell apoptosis, and the experiment was carried out according to the instructions of the kit. The cells were harvested and washed twice with PBS. Flow cytometry was performed using a BD FACSVerse flow cytometer, 10,000 cells were routinely collected, and the percentages of apoptotic Annexin V+ and necrotic PI+ cells among the total number of cells were analyzed using FlowJo 7.6 software.

### Live fluorescence of F-actin and microtubules

SiR-actin and SiR-tubulin were used to assess cytoskeletal changes in the entire injury therapy model. To make the staining solution, 1 μL of stock solution was diluted in 1 mL of cell culture medium. When the cells in the confocal Petri dish reached ~50% confluence, the culture medium was replaced with staining solution. The cells were placed in an incubator at 37 °C with 5% CO_2_ for 12 h and then imaged with a Zeiss LSM 800 confocal microscope. CellMask™ Deep Red Actin Tracking Stain was used to detect F-actin changes in the early stage of exosome treatment. To prepare the staining solution, 1 μL of stock solution was diluted in 1 mL of cell culture medium. The cells were incubated in staining solution for 1 h at 37 °C with 5% CO_2_ before exosome addition and imaged by a Leica TCS SP8 laser scanning confocal microscope.

### Immunostaining

The cells were fixed with 4% paraformaldehyde at room temperature for 10 min and then permeabilized with 0.1% Triton X-100 in PBS for 15 min. The Petri dish was washed with PBS and then blocked with 3% bovine serum albumin (BSA) at room temperature for 30 min. The cells were incubated with primary antibody at 4 °C overnight, washed with PBS and then labeled with secondary antibodies at room temperature for 1 h. The nuclei were stained with DAPI and the cells were imaged by a Leica TCS SP8 laser scanning confocal microscope.

### Statistical analysis

All of the experiments were performed at least three times. All quantitative data are displayed as the mean ± SD. *p* values were calculated by Student’s *t* test. Significance was measured at the following thresholds: N, no significant difference; *p < 0.05; **p < 0.01; and ***p < 0.001. All the data were processed in Origin (Origin 2019).

## Results and discussion

### Changes in cell traction force during MSC exosome therapy

**Fig. 1:**
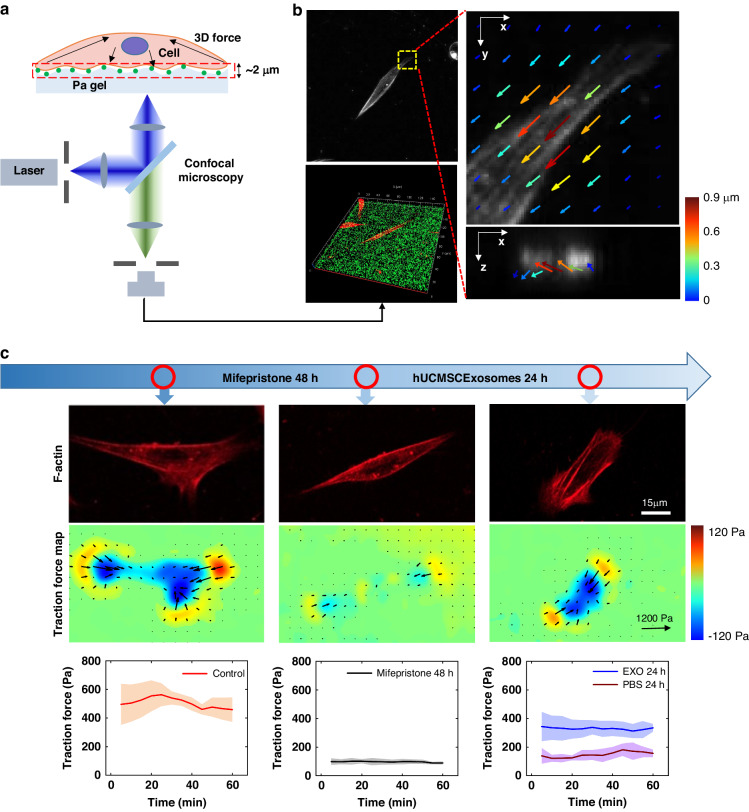
3D traction force microscopy platform and exosome therapy model. **a** Schematic of the design of cell traction force measurements with a single layer of fluorescent beads. **b** Schematic of a working image of the traction force microscopy platform. Live cell microfilament staining (red) and fluorescent particle (green) imaging were performed simultaneously. Corresponding calculated cell mechanical signals in and out of the plane are shown. The arrow color represents the magnitude of the displacement. **c** Schematic of MSC-derived exosome therapy models and the resulting 3D cell traction forces. The mean cell traction force of EndoSCs significantly increased with exosome treatment (*n* = 6)

Here, we used traction microscopy to characterize the therapeutic effects of stem cell exosomes on EndoSCs via mechanical signals. According to this method, cells were cultured on a gel substrate implanted with fluorescent beads, the displacement of the fluorescent beads was tracked by confocal microscopy to reflect the deformation of the gel substrate, and the mechanical interaction between the cells and the extracellular matrix was calculated. Since exosome therapy is a long-term process, to reduce the biological damage caused by long-term laser irradiation, we covered the substrate surface with a single layer of fluorescent beads to track the three-dimensional displacement of the substrate, which reduced the sampling time compared with the traditional method of embedding fluorescent beads in the gel. The substrate for the cell culture was mainly composed of PA hydrogels, with the scattering of fluorescent beads and modification of collagen type I on the surface (Fig. 1a, Supplementary Fig. 1a). The biocompatibility of PA hydrogels and the reliability of this fluorescent bead coupling were demonstrated in our previous work^25^. Since the deformation gradient of PA hydrogels is large, we improved the precision of the DVC results based on this monolayer fluorescent bead coupling method^27,34^. We selected a normally attached cell to demonstrate the cellular mechanical signal calculation process, in which cell microfilaments were fluorescently labeled, and both in-plane and out-of-plane mechanical signals can be measured (Fig. 1b). To characterize the ability of cells to exert mechanical forces on the substrate, we calculated the cell traction force (CTF) by averaging the absolute values of stress in the cell area, which was segmented by the cell profile.

To quantitatively characterize the force between EndoSCs and the substrate during treatment with exosomes, we constructed an injury model to simulate the damage state of the endometrium. Here, mifepristone was used as an injurious drug. One reason for using mifepristone as an injurious drug is that endometrial damage is usually caused by the overuse of contraceptives, and mifepristone is widely used as an emergency contraceptive. On the other hand, mifepristone can reduce progesterone and relieve pain and is commonly used in gynecological surgery. Here, we first cultured the cells on a gel substrate for 24 h to achieve stable adhesion, administered mifepristone for 48 h to simulate in vitro injury of EndoSCs, and then added exosomes for 24 h of treatment. The exosomes were extracted from the hUCMSC culture medium and purified at a concentration of 1.3 × 10^11^ particles/mL. For treatment, the exosomes were suspended in medium at a concentration of 1 µg/µL (Supplementary Fig. 2). We also optimized the stiffness of the hydrogel to improve its stability. Hydrogels with stiffnesses of 2.8 kPa, 8 kPa, 19 kPa, and 40 kPa were selected for EndoSC growth. The results showed that when the gel was soft (2.8 kPa), the adherent cells were more likely to fall off, and drug damage led to a sharp reduction in the number of cells used for experimental observation (Supplementary Fig. 3a). However, when the gel was hard (19 kPa and 40 kPa), the spread area and migration rate of the cells significantly increased, and the cells easily separated from the observation area (Supplementary Fig. 3b). In summary, a gel with a rigidity corresponding to 8 kPa in the literature was selected for the experiment^32^, and the actual calibration result of the rheometer was 5.8 kPa (Supplementary Fig. 1b). In particular, since the mifepristone and exosomes added during the experiment may bind to the substrate and affect its mechanical properties, a rheometer was also used to measure the stiffness of the gel treated with mifepristone and exosomes, and the results showed that the stiffness of the gel did not change significantly (Supplementary Fig. 3c).

Based on this model, we analyzed the CTF during the whole process. After 24 h of incubation of EndoSCs in a gel with a stiffness of 5.8 kPa, a CTF of ~600 Pa was produced (Fig. 1c). Moreover, the cells extended their pseudopods to all sides, showing a stable spreading pattern. After incubation with mifepristone for 48 h, the mechanical interaction between the cells and the outside matrix significantly decreased, and the CTF decreased to ~100–150 Pa. After removing the mifepristone and adding PBS for 24 h to establish the control group, we found only a small increase in traction force. For the group treated with MSC-derived exosomes for 24 h, we found that the CTF recovered to ~300–350 Pa, mainly due to the increase in in-plane mechanical signals. However, the traction force of the control group treated with PBS was still low, and the overall traction did not exceed 200 Pa. This suggests that the recovery of traction is due to exosome action. Accordingly, CTF can reflect the therapeutic effect of MSC-derived exosomes on cells. Moreover, we conducted mechanical signal detection for more than 1 h at each stage and found that the mechanical signal fluctuated little, proving the stability of this damage-treatment model and traction method.

### Exosomes improve cell motility and viability

**Fig. 2:**
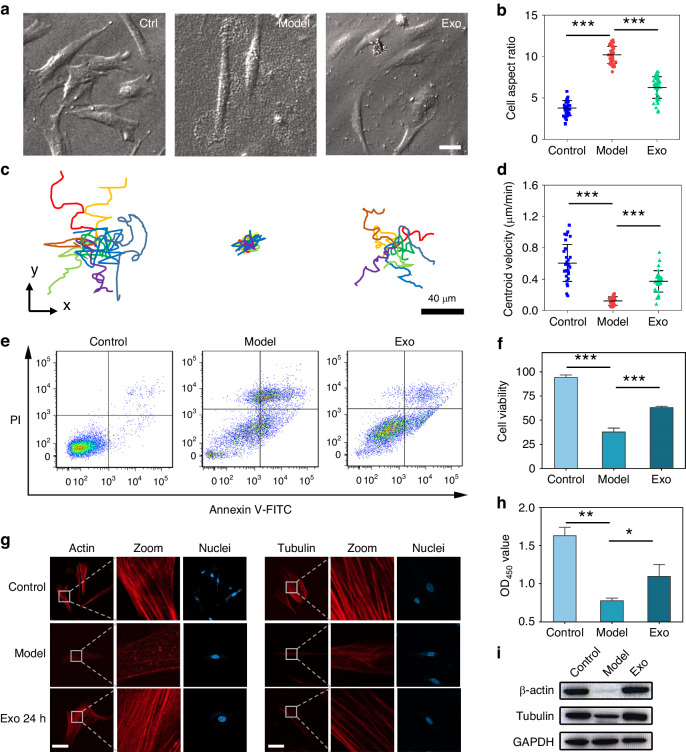
Effects of exosomes on cell motility and viability. **a** Changes in cell morphology during treatment. **b** Statistical results of (**a**) (n = 30). **c** Changes in the cell centroid during treatment (*n* = 8). **d** Statistical analysis of the cell centroid velocity (*n* = 30). **e** Flow cytometry results of the treatment model. **f** Cell viability test by flow cytometry (n = 3). **g** Living cell staining of the cytoskeleton of EndoSCs at different stages (scale bar: 40 µm). **h** Cell viability test by CCK-8 assay in this model (*n* = 3). **i** Western blotting analysis of the protein expression of β-actin and tubulin

To explore the effects of mifepristone injury and MSC exosome therapy on EndoSCs, we characterized the morphology and mobility of EndoSCs (Fig. 2a). Here, cell morphology was represented by the ratio of cell length to axis, and cell migration ability was represented by the distance of cell centroid movement. Mifepristone inhibited cell migration and caused the cells to stretch out in an elongated spindled state (Fig. 2b). After exosome treatment, the cell migration ability significantly improved, the cell morphology returned to normal with stable adhesion, and the cytoskeleton was restored (Fig. 2c, d). Cell polarity can reflect cytoskeletal structure and directional migration ability and is also associated with mechanical signals. During cell migration, it is usually necessary to polarize in the direction of migration and continuously extend the cell pseudopods. In our experiments, the cells lost their polarity after treatment with mifepristone, and the exosomes restored their polarity (Supplementary Fig. 4). This finding is consistent with the changes we observed in the cell migration ability.

To quantify the effect of exosomes on cells, we compared traditional biological methods, such as flow cytometry (FACS) (Fig. 2e, f) and CCK-8 cell activity assays (Fig. 2h). First, using flow cytometry with phosphotidylserine (PS) and Annexin V-FITC, the percentage of nonapoptotic cells increased from 33.9% to 63.8% after exosome treatment. This result indicated that the MSC-derived exosomes reduced mifepristone-induced EndoSC apoptosis. CCK-8 cell activity assays showed that cell activity was significantly greater in the exosome group than in the model group. These experimental data indicate that MSC-derived exosomes enhanced cell proliferation and inhibited cell apoptosis.

The mechanical signals applied by cells to the outside world are conducted by the cytoskeleton. The movement of myosin on the actin bundle forms the cell contraction force, and actin polymerization forms the cell protrusion force. Moreover, microtubules support the maintenance of the stability of the cell structure. Therefore, the influence on microfilaments and microtubules will be directly reflected in the CTF. To better understand the causes of CTF changes, the microfilaments and microtubules of cells were observed by live cell staining. We observed that the microfilaments and microtubules of the EndoSCs were uniformly arranged in the cell and presented a bright and clear bundle structure (Fig. 2g). After mifepristone injury, the fluorescence intensity of the microfilaments and microtubules decreased significantly. In addition, the overall fluorescence showed a scattered dot distribution, mainly distributed at the edge of the cell cortex; this indicated that mifepristone inhibited the polymerization of microfilaments and microtubules and made them exist in the cell as monomers. After exosome treatment for 24 h, the luminance of the fluorescence staining results clearly increased, and the microfilaments and microtubules showed a stable binding structure, which is very important for generating force on cells. Moreover, the western blotting results indicated that the protein levels of microfilaments and tubulin significantly decreased after mifepristone treatment and then recovered to normal levels after treatment with exosomes, which was consistent with the trend of the CTF we observed. The results of fluorescence staining verified the reparative effect of exosomes on the cytoskeleton and were in good agreement with the results of the western blotting assay (Fig. 2i). Intracellularly, cells maintain their stable morphology through a dynamic equilibrium between the contractile forces produced by microfilaments and the support provided by microtubules. Here, we observed that after treatment with exosomes, the number of microfilaments was notably greater than that of microtubules (Fig. 2g, i); this increased contractility accounts for the reduced cell elongation rate following exosome therapy (Fig. 2a, b).

To verify the general efficacy of exosomes and evaluate the broad applicability of the above mechanical approaches, we also investigated the direct effects of exosomes on three cell lines, including EndoSCs. The experimental results showed that without mifepristone treatment, the proliferation rate and migration ability of EndoSCs were also significantly improved after 24 h of exosome treatment, and the CTF was also significantly enhanced. In particular, after 48 h of exosome treatment, the cell proliferation rate still increased, but the CTF did not change significantly compared with that at 24 h, possibly because the CTF reached a relatively stable state (Supplementary Fig. 5). Human corneal epithelial cell-transformed (HCE-T) cells are often used in the study of eye diseases and respond to exosome therapy^36^. CCK-8 and scratch assays showed that compared with those in the control group, exosome treatment for 24 h significantly improved cell proliferation and migration, and the CTF was also significantly improved (Supplementary Fig. 6). MCF-10A cells are another type of normal human mammary epithelial cell, and our results showed that exosome treatment also led to improved CTF and promoted cell proliferation and migration (Supplementary Fig. 7).

### Real-time traction force detection in exosome therapy

**Fig. 3:**
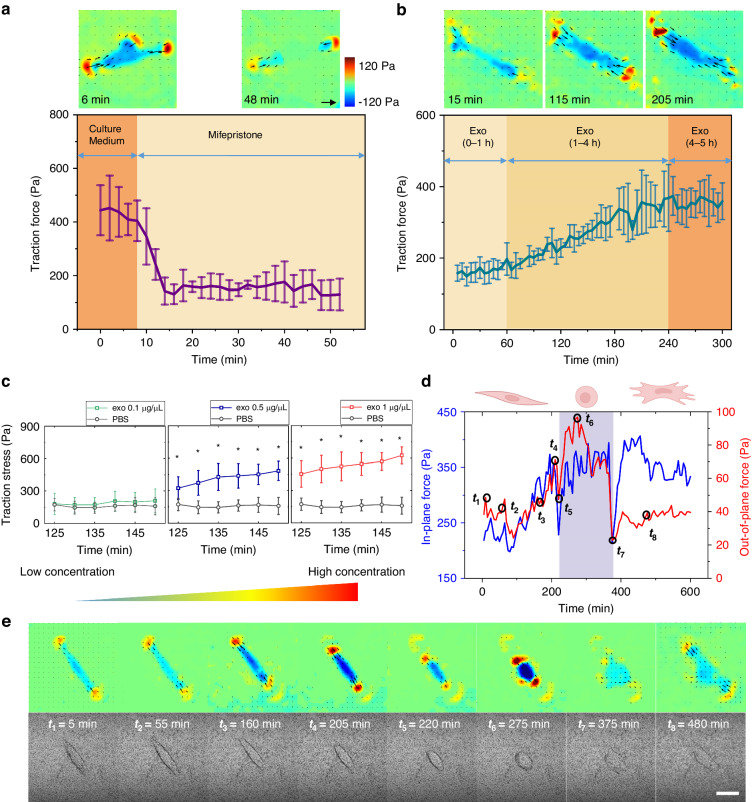
Real-time traction of exosome therapy. **a**, **b** Real-time cell mechanical signal detection during drug injury and exosome treatment (*n* = 4), after treatment with mifepristone (60 nM), and after treatment with MSC-derived exosomes (1 µg/µL). **c** The cell traction force of EndoSCs increased with increasing exosome concentration (*n* = 7, * represents a significant difference in traction force between the exosome group and PBS control group under the same treatment time, *p* < 0.05). **d** Statistical analysis of the cell traction force of a single EndoSC, which was captured every 5 min for 10 h after exosome treatment. **e** A series of 8 representative images of (**d**) (scale bar: 40 µm)

To investigate the early therapeutic effects of MSC-derived exosomes, we conducted real-time dynamic observations of CTFs generated by EndoSCs. First, we analyzed the CTF during the drug-induced injury process. The CTF was basically stable when the EndoSCs were cultured on the gel. With the addition of mifepristone, the traction force of the cells decreased rapidly. Within 6 min, the CTF decreased from 400 Pa to ~150 Pa, both in and out of the plane, and then stabilized without further decreasing (Supplementary Video 1). This shows that traction can sensitively reflect the stimulation of cells by drugs. We selected a stress map of a characteristic cell to demonstrate this process (Fig. 3a). With the action of mifepristone, the upper pseudopod of the cell gradually disappeared, and the cell adopted a symmetrical spindle state.

Then, we analyzed the dynamic mechanical signals induced by MSC-derived exosome therapy. In the early stage of exosome treatment (0–4 h), the overall traction force of the cells gradually increased (Fig. 3b). However, unlike that of mifepristone, the mechanical signal did not change immediately after the addition of exosomes. Through two-sample t tests, we found that the traction force significantly differed when the action time reached 1 h, before which the force increased relatively slowly. This might be due to the time elapsed during the process of the contents of exosomes entering cells and exerting their effects. Subsequently, the CTF increased significantly, from ~150 Pa at the beginning to ~400 Pa, and then reached a stable state (Supplementary Video 2). This increase was consistent with the performance of the mechanical signals in and out of plane, and the cell morphology changed from a symmetrical spindle to a spread state.

We also investigated the changes in cell traction force induced by different concentrations of exosomes. Since the traction of cells was significantly improved within ~60–180 min after the addition of exosomes, we first performed statistical analysis of the cell traction at 120 min after the addition of exosomes. A higher concentration of exosomes (1 µg/µL) promoted greater traction, while a lower concentration of exosomes (0.1 µg/µL) did not significantly increase the traction of cells compared with that in the control group treated with PBS. Moderate concentrations of exosomes (0.5 µg/µL) significantly increased the traction of cells, but the magnitude of increase was lower than that of high concentrations of exosomes (1 µg/µL) (Fig. 3c). These results showed that higher concentrations of exosomes in the early stages of action led to a stronger mechanical signal response. Therefore, we further compared its effects with those demonstrated in CCK-8 activity assays and flow cytometry analyses. We observed that cell viability and apoptosis did not significantly change in the early stages (2 h) of exosome treatment (Supplementary Fig. 8). This observation indicates that cell mechanical signals are more sensitive than cellular biological signals. In addition, we investigated the response of the cell traction force to the long-term effects (6 h, 24 h) of different concentrations of exosomes (Supplementary Fig. 9). For the experimental group with higher concentrations of exosomes (1 µg/µL and 0.5 µg/µL), the traction force still showed an increasing trend after 6 h of treatment. However, when the action time was further increased to 24 h, the traction force changed little compared with that at 6 h, and a small decrease even occurred. We believe that the cell traction force reached a relatively stable state. At 6 h, there was no significant difference in the traction force between the low-concentration group (0.1 µg/µL) and the control group. However, at 24 h and 48 h, the traction force significantly increased and then gradually increased. These results indicate that the enhancement effect of exosomes on traction force requires time, and there is an upper limit of saturation.

In addition, some cells showed more complex morphological changes during MSC exosome treatment. The cells quickly contracted and became round from the spindle-shaped state at the beginning and then spread out again after a period of time (bright field image in Fig. 3e, Supplementary Video 3). We selected one cell to represent this process (Fig. 3d, e). As shown in the figure, the cell morphology and traction remained relatively stable within ~1 h of exosome addition, at which time ${F}_{{xy}}$ (in-plane force) was 200 Pa and ${F}_{z}$ (out-of-plane force) was 40 Pa. Subsequently, the cells showed a significant increase in the out-of-plane and in-plane traction, and the cells also showed slight contraction. However, after ${t}_{4}$ = 205 min, when the traction had increased to ~ ${F}_{{xy}}$ = 350 Pa and ${F}_{z}$ = 70 Pa, the traction first appeared to rapidly decrease, reaching the lowest value within ~10 min. Then, the cells became round, along with a rapid increase in the out-of-plane and in-plane traction (t6 in Fig. 3d). The cells maintained a round state from t = 250 min to t = 350 min. At this time, the traction continued to fluctuate at ~ ${F}_{{xy}}$ = 350 Pa, which was similar to the in-plane traction force before the round stage. However, the out-plane traction was more unstable, and the maximum was ~ ${F}_{z}$ = 100 Pa, which was further enhanced compared with ${F}_{z}$ = 70 Pa before the round stage. After a certain period, the cells spread out again. During the spreading process, there was initially a significant decrease in traction force, which reached the lowest value in ~10 min. Then, the mechanical signal increased and finally reached a relatively stable state, where ${F}_{{xy}}$ = 350 Pa and ${F}_{z}$ = 40 Pa. Overall, exosome-induced enhanced cell traction oscillated and differed from the typical effects observed in drug treatment.

According to previous studies, cell rounding usually occurs in cases of cell division, apoptosis, and low adhesion^25,37,38^, in which cell traction is generally significantly reduced. In contrast, we found that the mean tractive force of the cells remained high and even increased when the cells were round, especially under out-of-plane stress. This abnormal mechanical phenomenon has attracted our attention, and this phenomenon is further described in the following section.

### Exosomes derived from MSCs induce short-term cell roundness

**Fig. 4:**
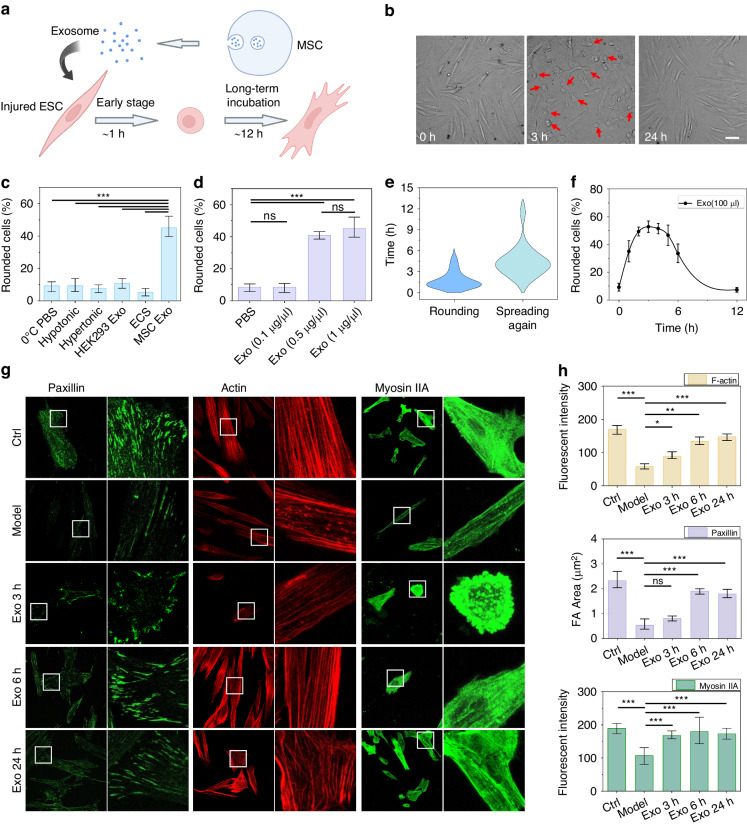
Early cell contraction and redevelopment after exosome treatment. **a**, **b** Schematic of the changes in cell morphology during exosome treatment. The red arrows indicate cells that became round in the early stage of exosome therapy. (scale bar: 50 µm). **c**, **d** The proportion of rounded cells under different conditions after treatment with exosomes for 3 h (*n* = 3). **e** The point at which a single cell became rounded and spread out (*n* = 31). **f** Changes in the proportion of rounded cells in a cell culture dish over time (*n* = 3). **g** Immunofluorescence staining results at specific time points during treatment (scale bar: 60 µm). **h** Statistical analysis of the FA area and fluorescence intensity in (**g**) (*n* = 11; 11 images from two independent experiments)

When MSC exosomes were applied to injured EndoSCs, we observed that the morphology of some cells exhibited a change in roundness, accompanied by an abnormal increase in cell traction force and subsequent expansion (Fig. 4a). As the rounding of EndoSCs occurred in the early stage of treatment with exosomes, we first focused on the possible physical factors that cause this cell rounding process. Studies have shown that temperature and osmotic pressure can affect cell morphology^39,40^. Although all of our previous experiments were performed at a live cell station and exosomes were rewarmed prior to their addition, temperature and osmotic pressure controls were added to study the factors contributing to cell roundness (45.3 ± 8.2%). In our experiments, 0 °C PBS or a hypertonic or hypotonic solution was added to the control group, and the proportion of rounded cells within 3 h after stimulation was determined (Fig. 4c). Only EndoSC cells treated with MSC-derived exosomes showed a large degree of roundness, indicating that osmotic pressure and temperature were not the causes of this phenomenon. When the cells appeared rounded after exosome treatment, membrane ruffles and blebs formed. Since the uptake of exosomes by cells involves membrane fusion, the instability of membrane tension may induce cell blebbing and lead to a transformation of cell morphology^41,42^. To study the effects of disturbances caused by exosome uptake in cells, we used exosomes extracted from 293 T cells as the control group, which did not show significant cell roundness. Since we extracted exosomes with a kit, we established a control group using medium without stem cells and the same exosome extraction process to exclude interference from the kit buffer. Again, there was no significant cell roundness. The above experimental results indicate that the contents of MSC-derived exosomes mainly mediate this phenomenon (Supplementary Fig. 10). Further analysis of the effects of different concentrations of exosomes (Fig. 4d) revealed that the proportion of rounded cells did not change significantly at lower exosome concentrations (0.1 µg/µL) but increased significantly at higher exosome concentrations (0.5 µg/µL and 1 µg/µL). Moreover, the ratio of rounded cells to high-concentration exosomes (0.5 µg/µL, 1 µg/µL) was basically the same, suggesting that there may be a threshold screening process for the generation of the contraction roundness phenomenon. The exosome concentration corresponding to this cell roundness is similar to the exosome concentration corresponding to the traction force response, which indicates that cell roundness may be related to increased cell traction force. We also analyzed the time points at which individual cells contracted and spread after treatment with exosomes, as well as the proportion of rounded cells in the Petri dish over time (Fig. 4e, f). The beginning of cell roundness was mainly concentrated in the first 3 h, while the respreading stage was mainly concentrated in the first 2–6 h, and the proportion of rounded cells also showed a downward trend at 6 h. At 12 h, the proportion of rounded cells decreased to the level observed before exosome treatment (Supplementary Fig. 11, Supplementary Video 4).

After statistical analysis of the cell rounding phenomenon, we found that 3 h and 6 h after exosome addition were appropriate time points for observing cell contraction and spread. The stability of the cell morphology is actually the result of the coordination of the cell contraction force and cell adhesion force^38^. For example, the increase in cell contractility induced by myosin will lead to cell contraction; microfilament and microtubule polymerization will lead to the protrusion of cell pseudopods and promote cell spreading; and cell focal adhesion (FA) depolymerization will lead to decreased cell adhesion and cell rounding. To further study the role of mechanical signals in the cell rounding process, several proteins, including paxillin, myosin2a and F-actin, were selected for immunofluorescence staining to characterize cell adhesion, cell contraction and the actin cytoskeleton (Fig. 4g). As shown in Fig. 4g, compared with normal cells, paxillin showed a darker spot-like distribution after the addition of mifepristone for 48 h, indicating that mifepristone inhibited the normal adhesion of cells and basically did not form mature FAs. At the same time, actin was concentrated in the cortical area of the cells, and shorter stress fibers (SFs) were scattered in the middle area of the cells. The fluorescence intensity of myosin IIA decreased, and myosin IIA was also inhibited. In general, after 48 h of mifepristone treatment, the cytoskeleton was damaged, and the adhesion between cells and the substrate was weak, which might be the cause of the low traction. After mifepristone was removed and exosomes were added for 3 h, the cells exhibited marked contraction and roundness. At this time, we found that the intensity of F-actin remained relatively low, but that of myosin2a showed obvious enhancement. The fluorescence intensity of paxillin was significantly improved, indicating the formation of adhesive patches, but the areas were generally small and did not form mature adhesion spots. After the addition of MSC exosomes for 6 h, most of the cells returned to a stable adhesion state, and the number of mature paxillin adhesion sites significantly increased. Moreover, actin and myosin2a were highly expressed. At this point, the cell spread area was significantly increased, greater cell traction was generated, and the cytoskeleton damage caused by mifepristone was repaired, with reforming stable dense actin bundles. After 24 h of exosome treatment, the intensity of microfilament fluorescence staining further increased and was close to that of the fresh cells, indicating that the cell state and structure were close to those of normal cells, which explains why the cells could produce greater mechanical signals after 24 h of exosome treatment. In conclusion, MSC-derived exosomes enhanced the number of microfilaments, myosin, and adhesion sites on EndoSCs damaged by mifepristone (Fig. 4h). Thus, the earlier significant enhancement of myosin relative to that of the microfilaments and adhesion plaques may be the factor leading to cell roundness.

### Mechanical and biological mechanisms of rapid morphological transformation

**Fig. 5:**
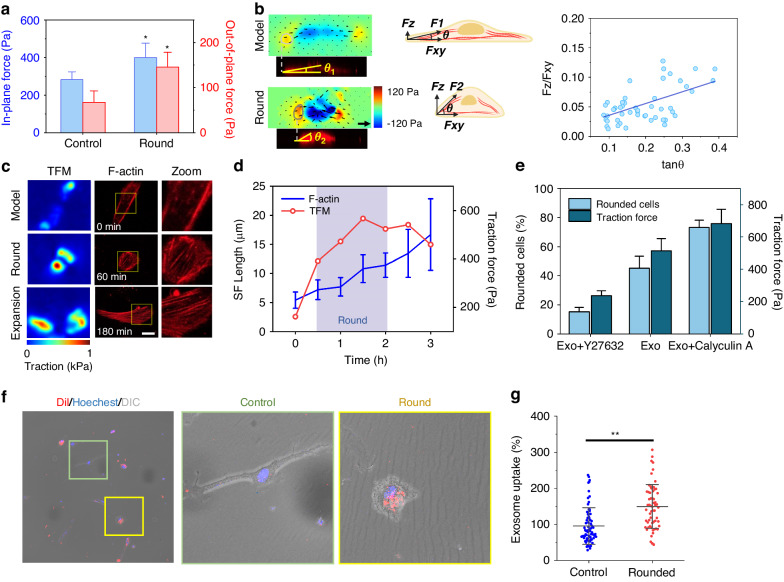
Cytoskeletal changes and exosome uptake during cell roundness. **a** In-plane and out-of-plane forces of different cell morphologies incubated with exosomes for 3 h (*n* = 7). **b** The inclination of traction at the front end of the long axis of cells is correlated with the inclination of the cytoskeleton (statistical results of 7 cells). **c** F-actin live-cell staining and traction force map of a representative cell (scale bar: 20 µm). **d** The changes in traction force and stress fiber length in the representative cell. **e** After treatment with 50 µM Y27632 or 100 nM calyculin A in combination with exosomes for 3 h, the fraction of rounded cells was compared with the traction force in EndoSCs (*n* = 87 and *n* = 6, respectively). **f**, **g** Confocal images of EndoSCs incubated with DiI-labeled exosomes at 37 °C for 3 h. The exosome uptake of the round and control groups was quantified by determining the fluorescence intensity (scale bar: 70 µm) (9 fluorescence images from three independent repeat experiments)

CTFs are generated by myosin interactions and actin polymerization and are then transmitted to the outer matrix through adhesion. To further explore the relationship between mechanical signal changes and cytoskeletal dynamics during MSC exosome therapy, we conducted traction force measurements concurrently with live-cell F-actin staining. Our initial findings revealed that cells with a rounded morphology exhibited greater traction forces, with out-of-plane stress demonstrating a more pronounced response than in-plane stress (Fig. 5a). Here, we analyzed microfilament-stained images to determine the angle between the cytoskeleton and the substrate. Remarkably, we observed a positive correlation between the ratio of out-of-plane force to in-plane force at the cell periphery and the inclination of actin cytoskeletal organization (Fig. 5b, Supplementary Fig. 12). This indicates that the orientation of traction force is closely related to cell morphology, with the increase in cytoskeleton inclination caused by cell roundness being the main reason for the rapid increase in out-of-plane force.

We also monitored changes in the actin cytoskeleton during the initial phases of exosome action, focusing our discussion on a representative cell (Fig. 5c, Supplementary Fig. 13). When exosomes were added, the cells initially assumed a rounded morphology, accompanied by a rapid increase in traction force. At this stage, the SF length remained relatively short, and the SF orientation was dispersed. Subsequently, as the cells resumed spreading, there was a notable increase in both the quantity and length of SFs, with their alignment becoming largely parallel to the direction of the traction force (Fig. 5d, Supplementary Video 5). These observations suggest that enhanced cell traction plays an important role in driving stress fiber formation and that this enhancement of mechanical signaling occurs at an early stage, preceding the maturation of the microfilament cytoskeleton.

In fact, we observed that the roundness of the cell was basically accompanied by an increase in traction force, and our earlier staining results also indicated a significant enrichment of myosin near the cortex of rounded cells (Figs. 3d, 4g). We hypothesize that this myosin activation leads to enhanced contractility, which is the primary driver of cell roundness and the subsequent increase in traction force. To confirm this hypothesis, we treated injured cells with appropriate doses of calyculin A or Y27632 in combination with exosomes. Calyculin A promoted myosin activity, whereas Y27632 had an inhibitory effect^43,44^. The results showed that calyculin A increased the proportion of rounded cells to 73%, with a concurrent substantial increase in cell traction force (Fig. 5e, Supplementary Fig. 14). Conversely, treatment with Y27632 significantly inhibited the formation of rounded cells, resulting in relatively low traction force. Together, these findings suggest that cell roundness in the early stages of exosome action is due to an increase in cell contractility.

The above experiments show that cell traction can reflect the therapeutic effect of exosomes as a sensitive and effective method and higher concentrations of exosomes lead to greater cell contractility (Fig. 3c). Here, we found that rounded cells produced greater cell traction, which also reflects the possibility that rounded cells are subject to stronger therapeutic effects from exosomes (Fig. 5a). To investigate the association between cell roundness and exosomes, we labeled exosomes with the lipophilic dye Dil and quantified the process of exosome uptake by confocal microscopy (Supplementary Fig. 15). As shown in Fig. 5f, when the EndoSCs were incubated with exosomes for 3 h, the rounded cells contained more labeled exosomes, and the statistical results showed that the rounded cells had a greater exosome uptake capacity (Fig. 5g). These results further demonstrate that early transient cell rounding was caused by exosome action. Exosome uptake is influenced by cellular endocytosis mediated by multiple pathways, in which macropinocytosis is regulated by Rho family GTPases^45^. In addition, cell rounding, rapid traction force enhancement, and cytoskeletal recombination during early exosome treatment are also associated with Rho family GTPase activation^46^, suggesting that exosomes may mediate the expression of Rho GTPases in EndoSCs.

**Fig. 6:**
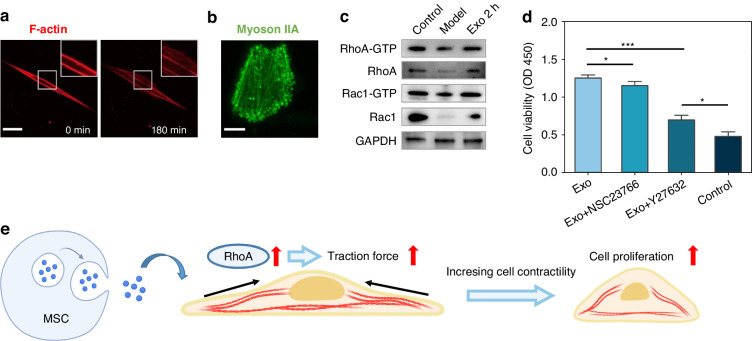
Exosomes induce the activation of Rac/RhoA in EndoSCs. **a** F-actin live-cell staining showed lamellar pseudopodia (red) in the early stage of exosome therapy (scale bar: 30 µm). **b** Myosin IIA immunofluorescence staining (green) revealed cell blebbing when the cell was rounded, and the bleb appeared to be enriched with myosin IIA (scale bar: 10 µm). **c** western blot and pull-down assays showed that the expression levels of RhoA, RhoA-GTP, Rac1, and Rac1-GTP were elevated in the MSC-Exo-treated EndoSCs. **d** The effects of the Rhoa inhibitor Y237632 and the Rac1 inhibitor NSC23766 on cell proliferation during exosome treatment (*n* = 3). **e** Exosomes mediate the activation of RhoA in EndoSCs, thereby promoting cell proliferation. This early effect is concurrent with an increase in traction force

Rho GTPases can regulate cell mechanics-related behaviors as a family of proteins, among which RhoA and Rac1, two major subtypes, control cell contraction and the formation of cellular pseudopods, respectively, to affect cell morphology, in addition to causing cell phenotypic transformation^47–49^. In our previously constructed cell damage therapy model, MSC exosomes also caused the formation of lamellar pseudopods and cell blebs, which may be mediated by the effect of exosomes on Rac1 and RhoA protein expression in EndoSCs (Fig. 6a, b). To verify this hypothesis, we tested the expression of Rho GTPase in the injury model. A pull-down assay and western blot analysis showed that the expression levels of total Rac1, Rac1-GTP, RhoA, and RhoA-GTP were elevated in EndoSCs treated with exosomes for 2 h (Fig. 6c). This finding also confirms that the interaction between exosomes and recipient cells occurs at a relatively early stage. To further determine the roles of Rac1 and RhoA in exosome-mediated cell proliferation, we used the RhoA inhibitor Y27632 and the Rac1 inhibitor NSC23766 to downregulate the expression of RhoA and Rac1, respectively. Upon evaluating the cell proliferation, we observed that both Y27632 and NSC23766 exerted inhibitory effects, with Y27632 demonstrating a more pronounced impact (Fig. 6d). These findings indicate that RhoA signaling plays a critical role in exosome-mediated cell proliferation.

In the literature, numerous studies have shown that RhoA activity is closely related to cell proliferation^50^. Mitotic cell rounding occurs during mitosis when a flat interphase cell transitions into a spherical shape, which is important for the proper separation of chromosomes and the normal progression of mitosis^51^. Mitotic cell rounding is accompanied by changes in the actin cytoskeleton and FAs^52,53^. In particular, a prior investigation emphasized the indispensable role of Rho-kinase in mitotic cortical retraction and stiffening^54^. This previous study showed that the activation of RhoA induces an increase in cell contractility, leading to the reorganization of the actin cytoskeleton and resulting in mitotic cell rounding. In our experiments, we observed similar phenomena in EndoSCs treated with exosomes. This suggests that exosome therapy enhances the contractility of injured cells, promoting reorganization of the cellular cytoskeleton and thereby facilitating the progression of mitosis.

Overall, these results suggest that MSC-derived exosomes promote the expression and activation of the RhoA protein in EndoSCs, resulting in enhanced cell contractility, which ultimately manifests as an increase in traction and cell roundness (Fig. 6e). We further investigated the role of cell contractility during exosome therapy through drug treatment, and when we inhibited cell contractility, exosome-induced cell proliferation was significantly reduced, approaching the level of untreated cells. In fact, numerous studies have shown that cell contractility is a key factor affecting the roundness of mitotic cells. Here, we propose that exosomes enable damaged cells to regain their contractile ability, enabling them to undergo mitosis normally.

## Conclusion

In summary, this study revealed that cell traction forces are highly intuitive and can be used to rapidly quantify the enhancement of cell contractility induced by MSC-derived exosomes, thereby characterizing the therapeutic effectiveness of exosomes. Moreover, we observed that in the early stages of exosome action, there is a rapid increase in the cell traction force, accompanied by rapid changes in cell morphology. The results showed that cell mechanical signal monitoring was more sensitive than cell viability (CCK-8 assays) and apoptosis (Annexin V-FITC/PI) detection. Based on the results of fluorescence staining, we verified the enhancement effect of exosomes on the cytoskeleton, and by labeling exosomes, we confirmed that the effect of exosomes depends on the number of exosomes uptake. Further experiments confirmed that MSC-derived exosomes promoted cell contraction by enhancing the activation of RhoA in recipient cells, inducing reorganization of the cytoskeleton and consequently facilitating cell proliferation. The results demonstrate that we can quickly evaluate the early therapeutic effects of exosomes by analyzing the mechanical signals of cells. This can accelerate both exosome research and clinical applications, contributing to a better understanding of the molecular mechanisms through which exosomes exert their therapeutic effects.

### Ethics declarations

hUCMSCs were isolated from human umbilical cord tissues and the EndoSCs were isolated from human endometrial tissue. The volunteer consented to donate and provided informed consent. The protocol was approved by the Ethical Committee of Anhui Medical University (No. 20190438).

### Supplementary information

Revised Supplementary materials-clean version

Supplementary Video 1

Supplementary Video 2

Supplementary Video 3

Supplementary Video 4

Supplementary Video 5
