# Supplementary material for: Dynamic nanomechanical characterization of cells in exosome therapy
Source: Microsyst Nanoeng. 2024 Jul 15;10:97. doi: 10.1038/s41378-024-00735-z (PMC11251037; doi:10.1038/s41378-024-00735-z)
Supplement: Supplementary file 1 — Revised Supplementary materials-clean version [file 41378_2024_735_MOESM1_ESM.docx]

**Supplemental information**

**
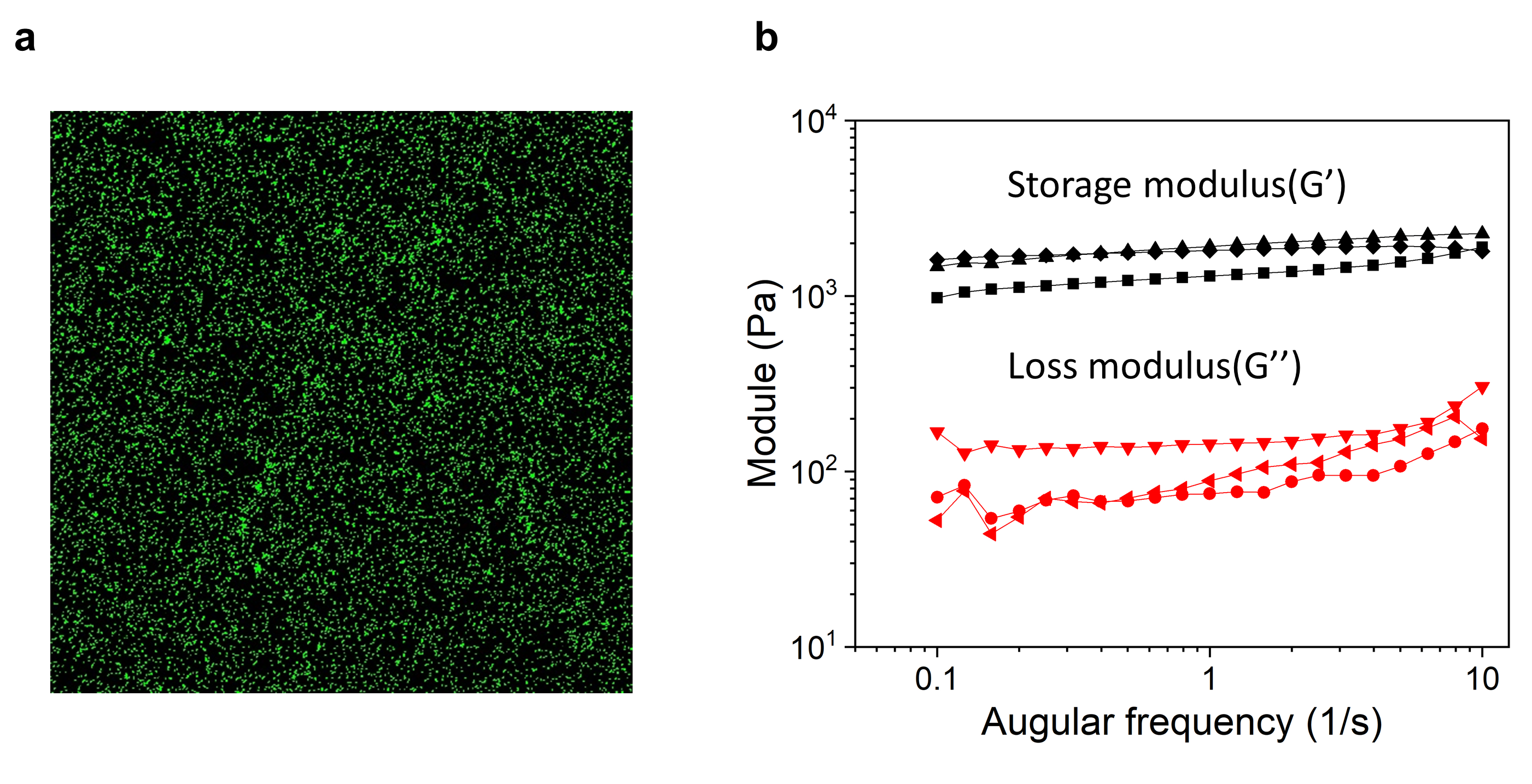
**

**Supplementary Fig. 1. a,** Confocal fluorescent images of fluorescent beads in PAA gels. The size was 160 μm × 160 μm and 512 pixels × 512 pixels. **b,** Dynamic frequency sweep tests of PAA storage modulus and loss modulus (the experiment was repeated three times independently), the frequency changed from 0.1 rad/s to 10 rad/s and Young’s modulus (E=2(1+ν)G') was calculated to be about 5.8 kPa.


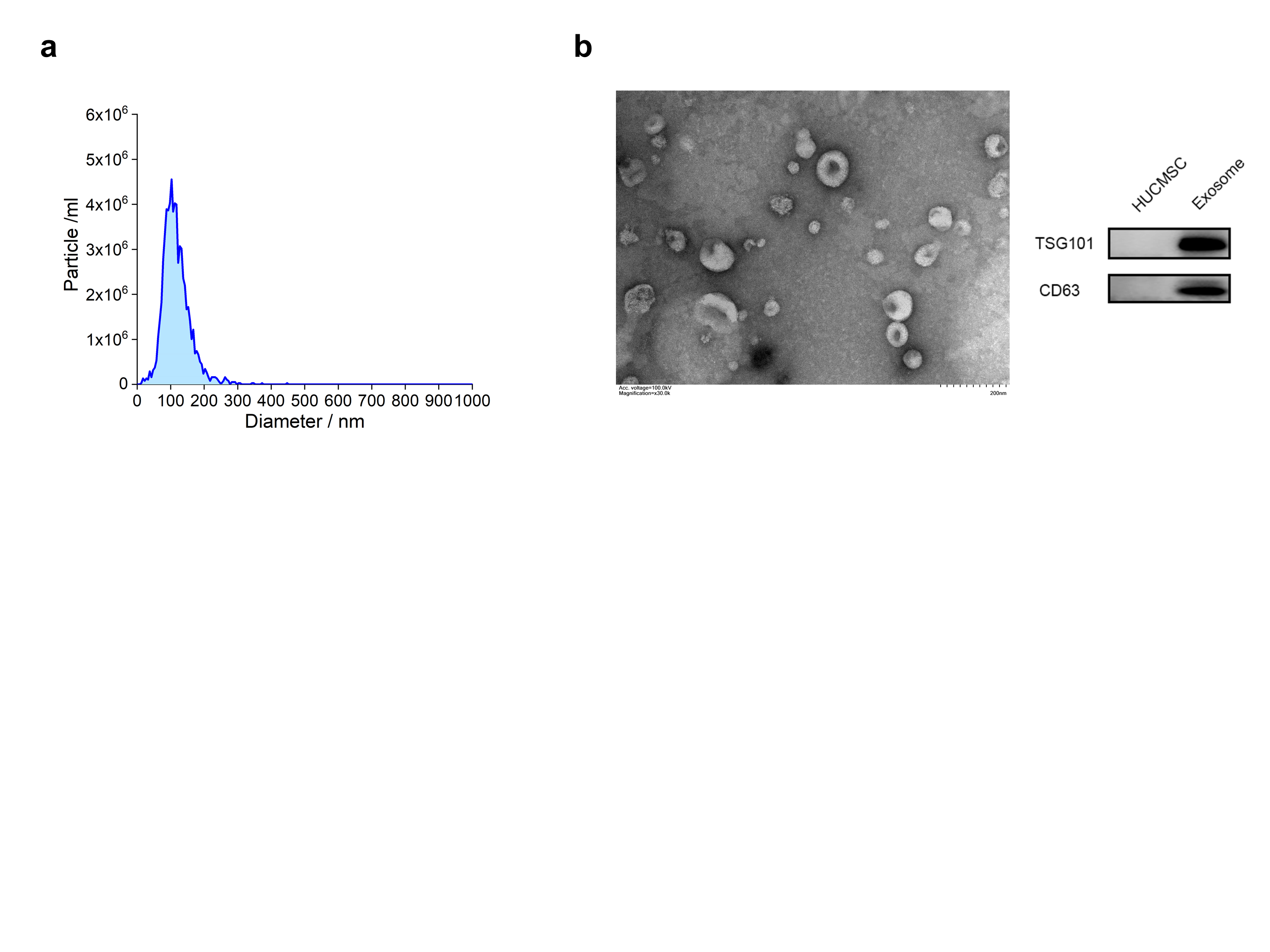


**Supplementary Fig. 2. a,** Size distribution of purified MSC exosomes, the diameter peak was about 100 nm, as the sample was diluted 2000 times for testing, the concentration was about 1.3 × 10^11^ particles/mL. **b,** Exosome morphology was characterized by electron microscopy and exosome surface characteristic proteins were characterized by Western blot.


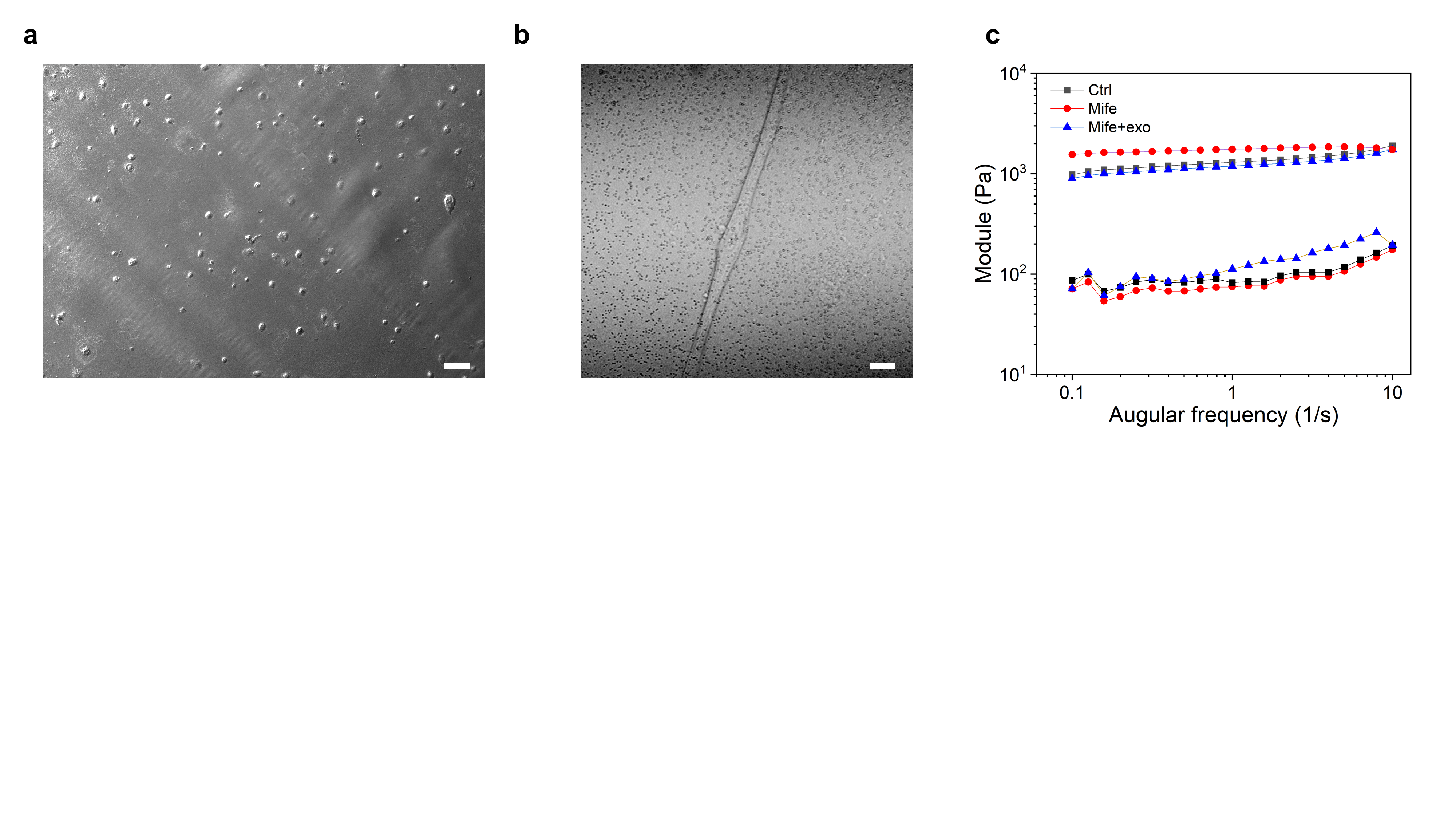


**Supplementary Fig. 3. a,** On the gel with a stiffness of 2.8kpa, EndoSCs were basically deglued and spherical after drug injury (scale bar:40 μm). **b,** EndoSCs spread over a large area on a gel with a stiffness of 19kpa. When the objective lens is 40X, the displacement calculation accuracy is higher, but the cells are easily to exceed the area of observation (scale bar:10 μm). **c,** Measurement results of gel mechanical properties corresponding to different time periods during the experiment.


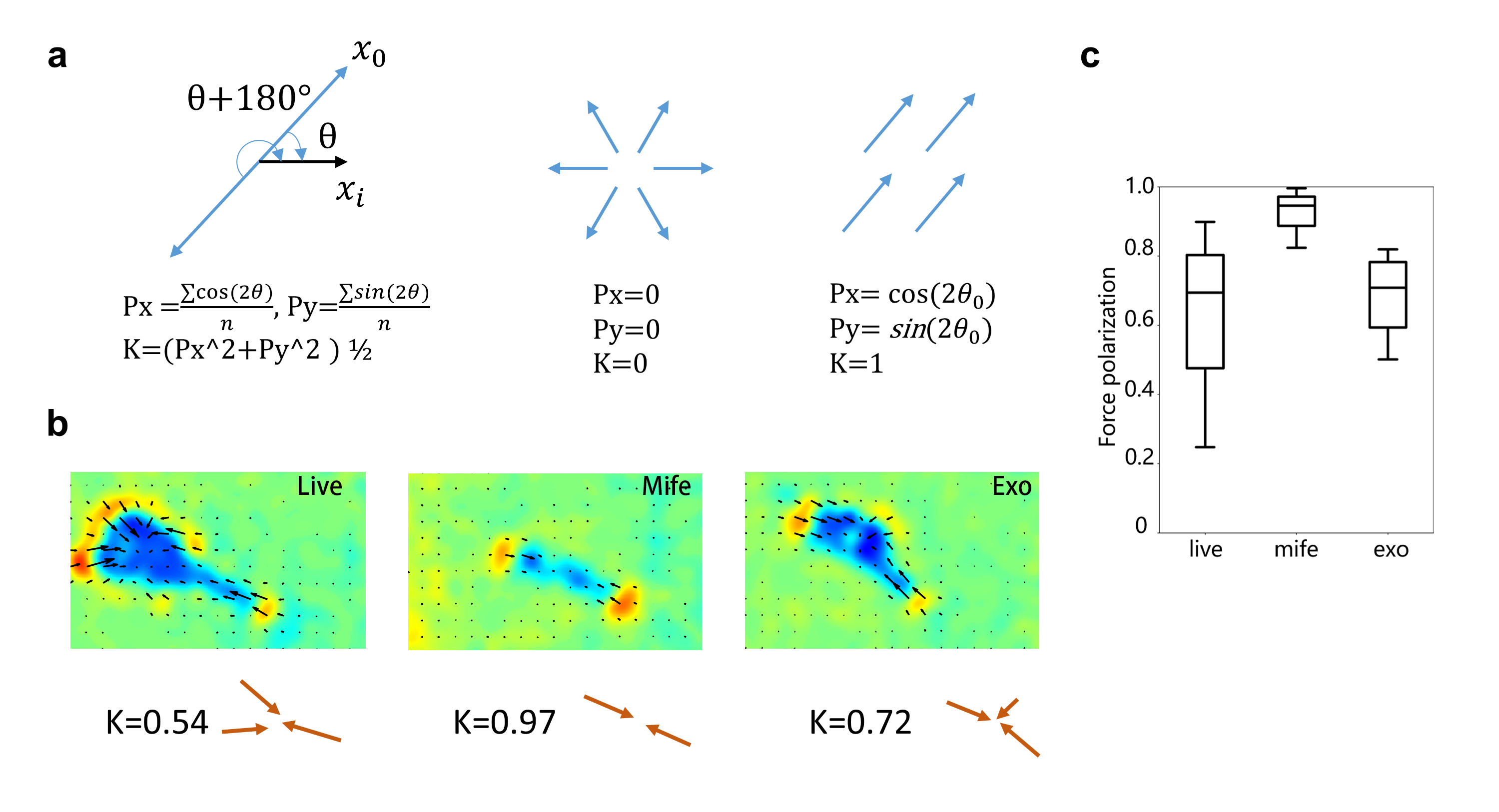


**Supplementary Fig. 4.a,** Calculation of the cell traction force polarization. For a traction force map, first we establish the principal axis of traction, which we assume here is $x_{0}$. For a definite $x_{0}$, we can measure its relation to all traction forces by the value of K. If the direction of the traction force diverges, K=0, and the direction is the same, K=1. **b,** Representative cell polarity results at different stages of exosome therapy models. **c,** Statistic results of different stages (n=30).


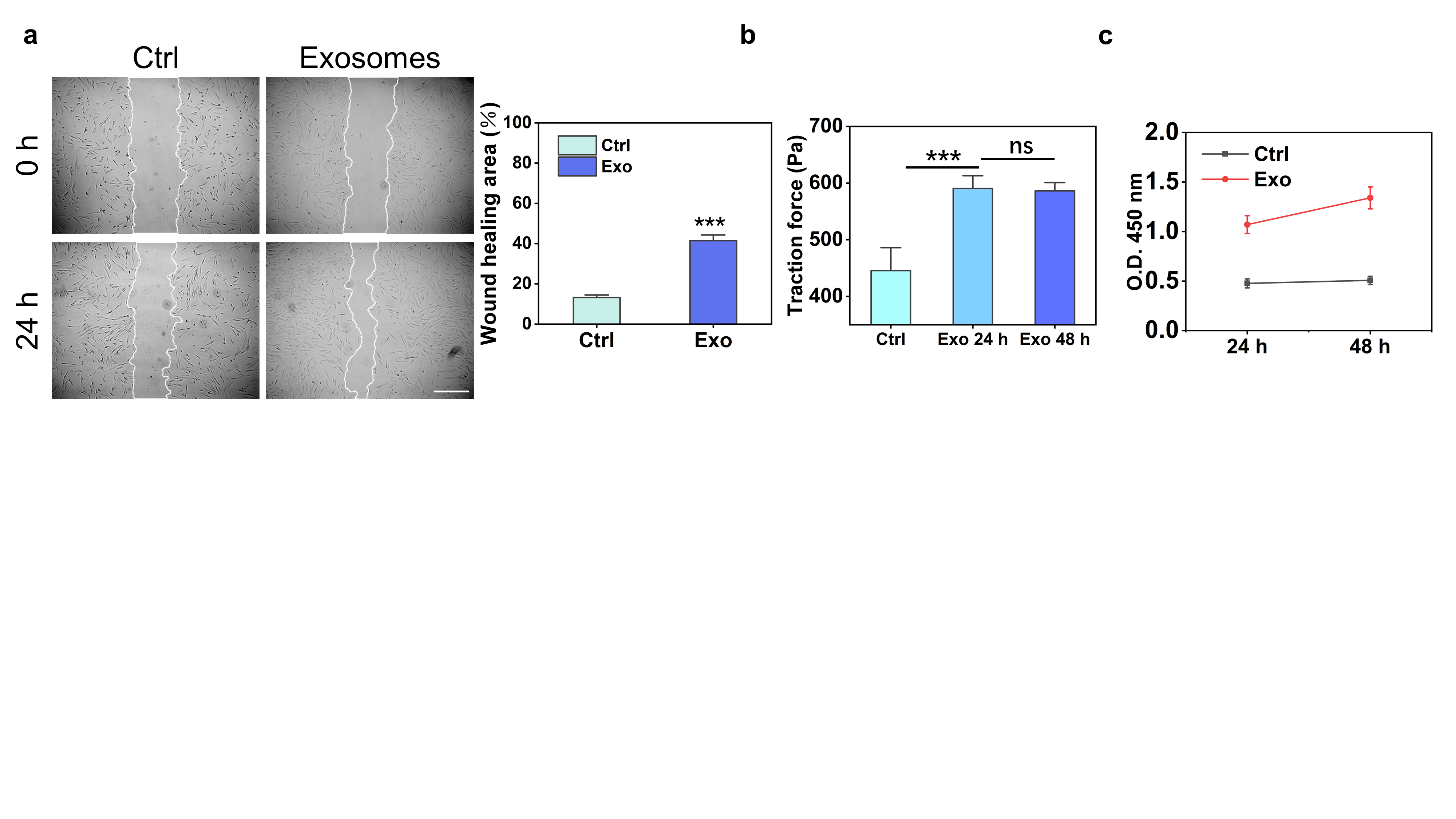


**Supplementary Fig. 5.a,** Representative images of the scratch wound healing assay for EndosSCs showing that the wound healing rate in the Exosomes group was higher at 24 h than that in the control group (n=3). Scale bar = 200 μm. **b,** Cell traction force of EndoSCs increased when treated with exosomes for 24 h. However, when further applied to 48h, the cell traction force basically did not change (n=6). **c,** A CCK-8 assay showed that exosomes treatment had significant effect on the proliferation rate of EndoSCs compared with the control group at 24 h and 48 h (n=3).


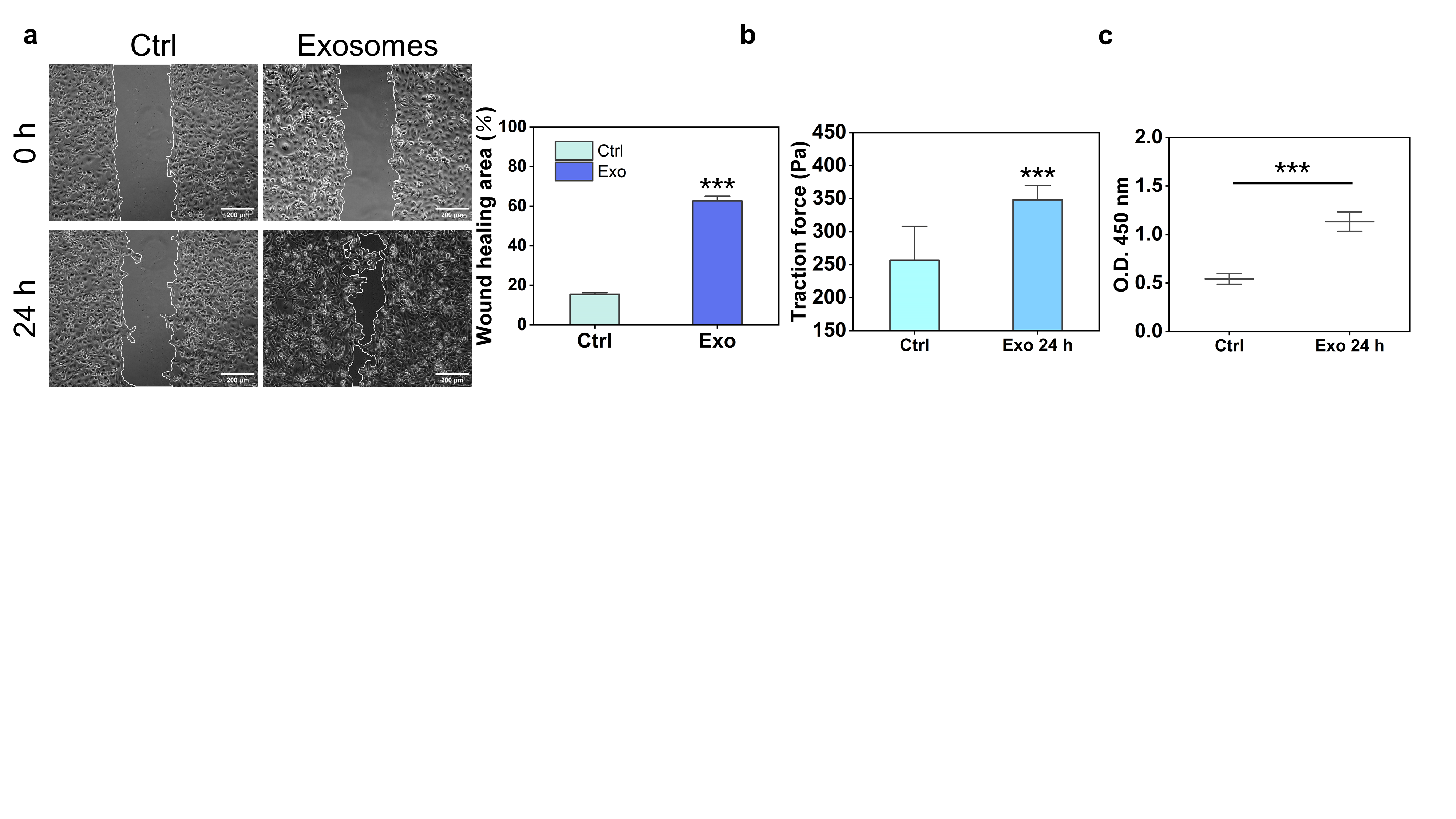


**Supplementary Fig. 6.a,** Representative images of the scratch wound healing assay for HCE-T cells showing that the wound healing rate in the Exosomes group was higher at 24 h than that in the control group (n=3). Scale bar = 200 μm. **b,** Cell traction force of HCE-T cells increased when treated with exosomes for 24 h (n=6). **c,** A CCK-8 assay showed that exosomes treatment had significant effect on the proliferation rate of HCE-T cells compared with the control group at 24 h (n=3).


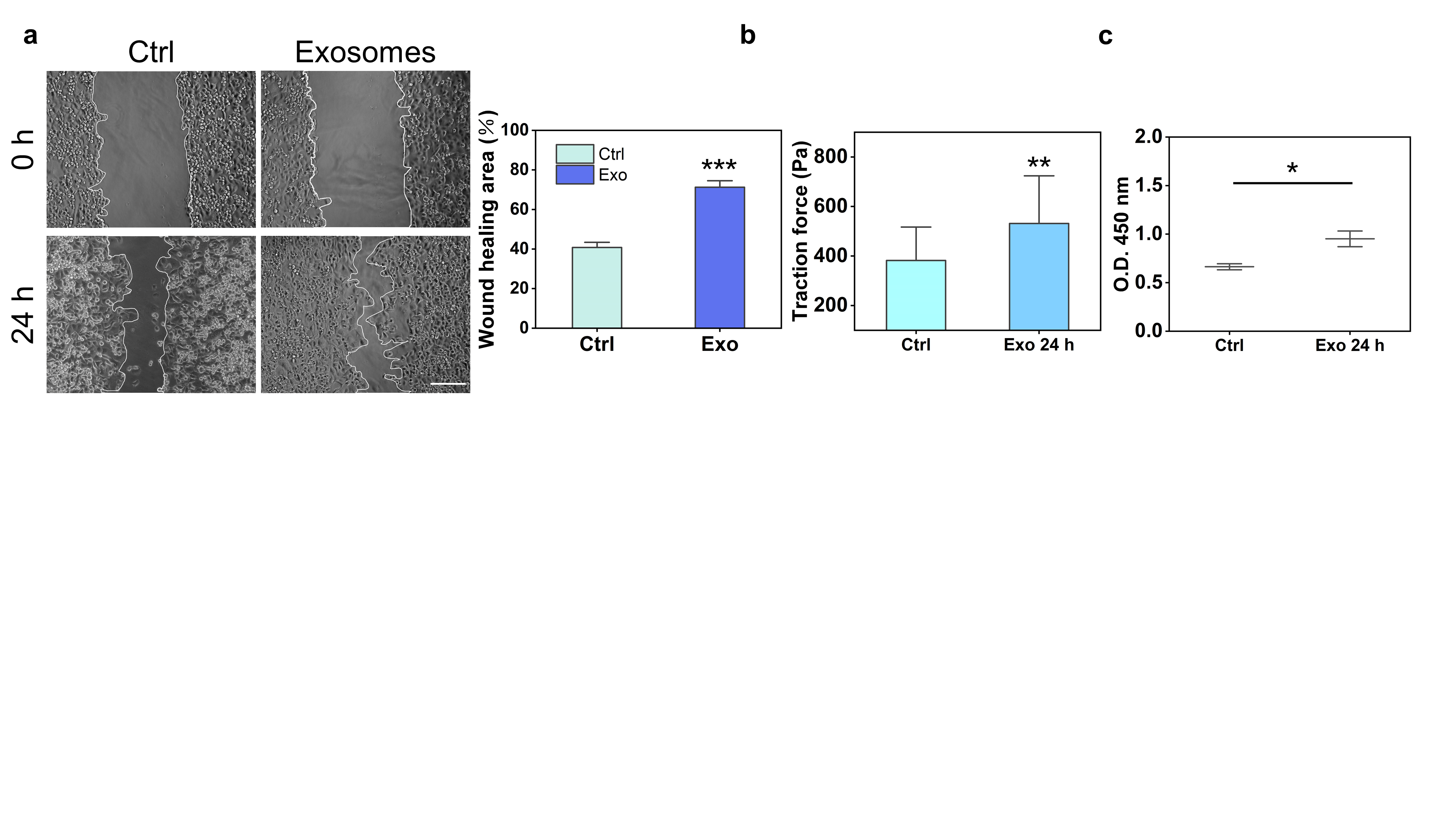


**Supplementary Fig. 7.a,** Representative images of the scratch wound healing assay for MCF-10A cells showing that the wound healing rate in the Exosomes group was higher at 24 h than that in the control group (n=3). Scale bar = 200 μm. **b,** Cell traction force of MCF-10A cells increased when treated with exosomes for 24 h (n=6). **c,** A CCK-8 assay showed that exosomes treatment had significant effect on the proliferation rate of MCF-10A cells compared with the control group at 24 h (n=3).


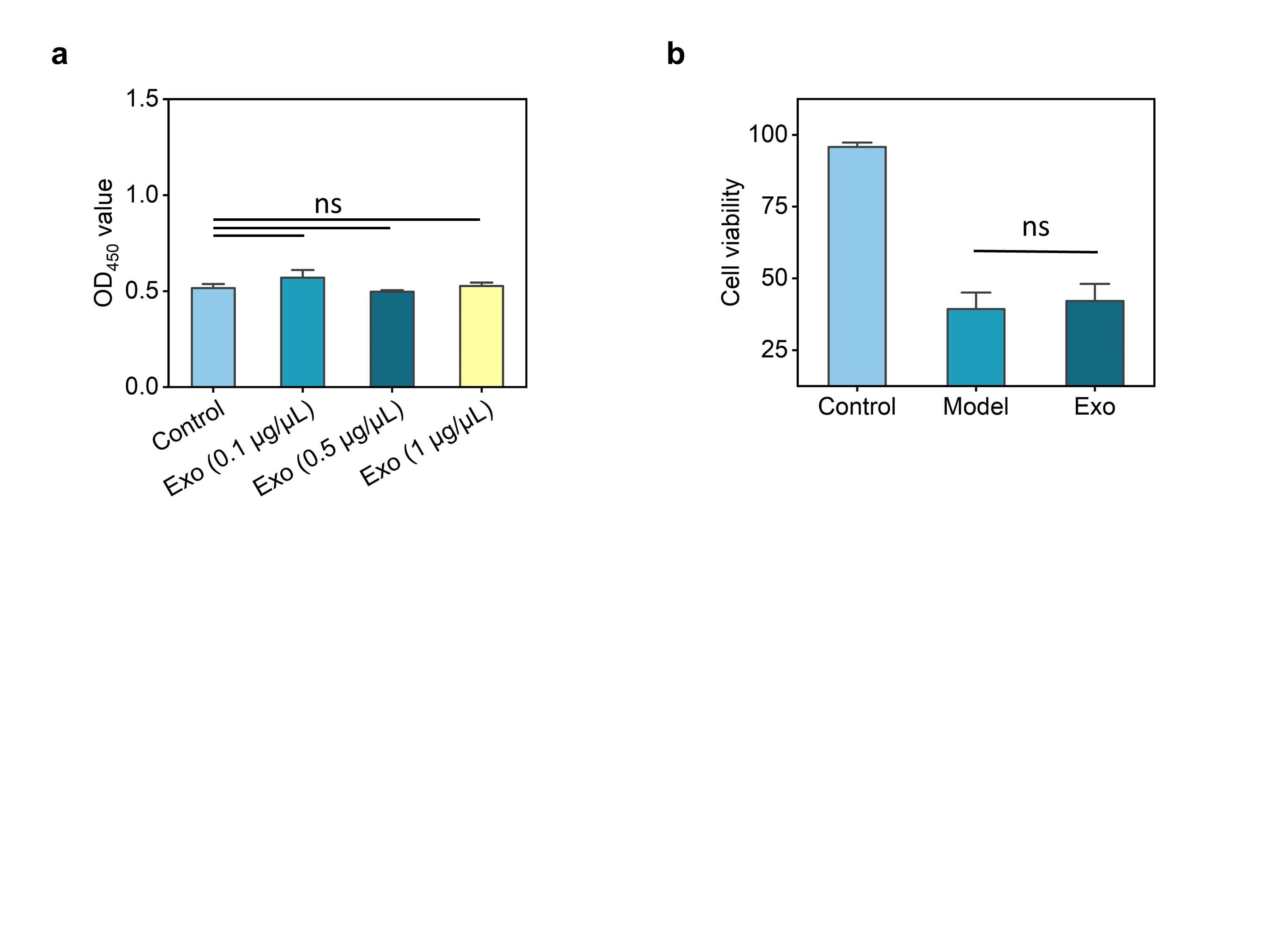


**Supplementary Fig. 8.a,** Cell viability test by CCK-8 assays when treated with exosomes for 2 h, the control group treated with PBS. **b,** Flow cytometry results of the treatment model when treated with exosomes for 2 h, indicating that apoptosis is not significantly improved in the early stage of exosomes action.


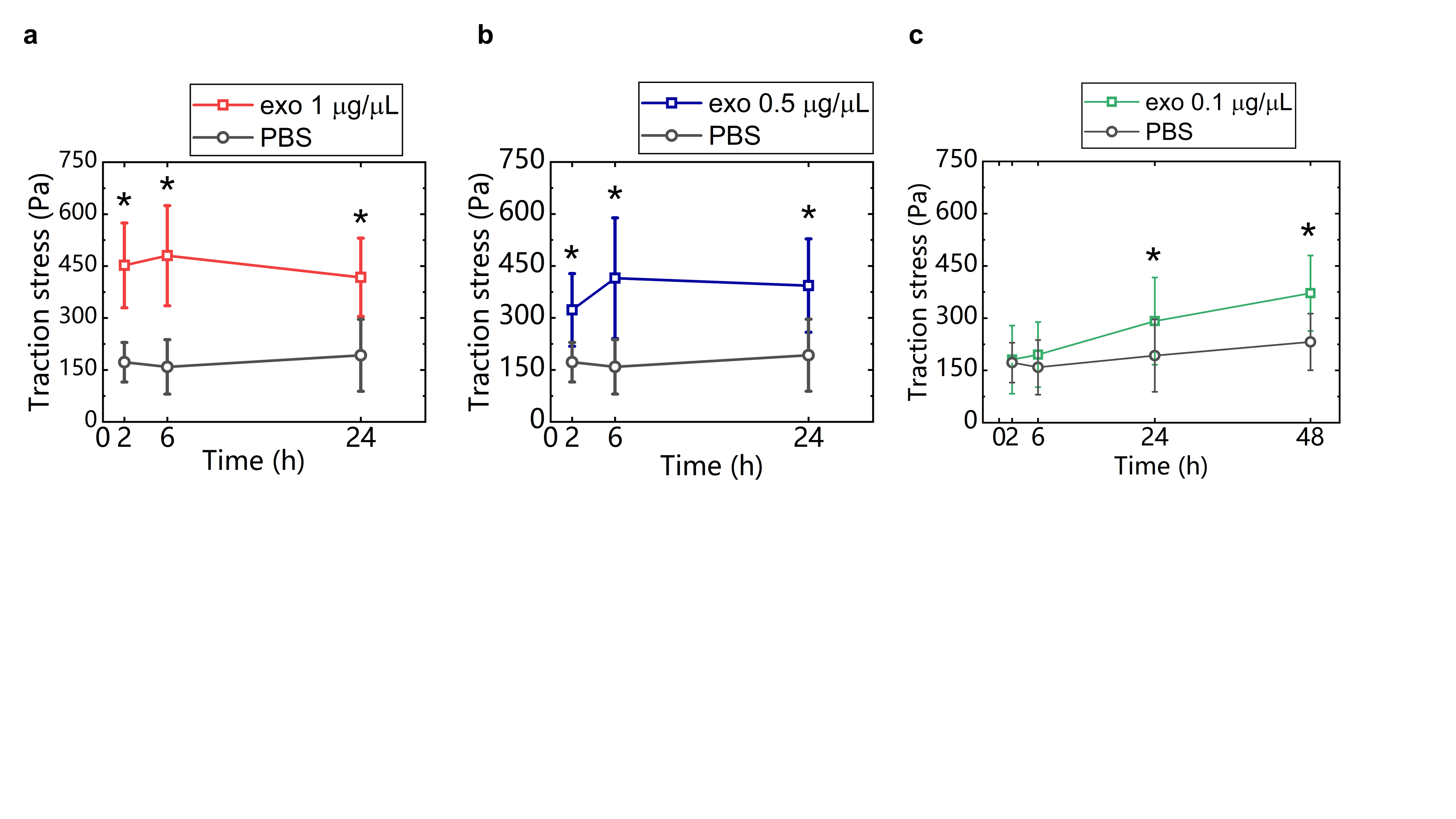


**Supplementary Fig. 9.** Mechanical signal response of EndoSCs when treated with exosomes of different concentrations (**a,** 1 µg/µL, **b,** 0.5 µg/µL, **c,** 0.1 µg/µL). (n=6, * represents the statistical differences in traction force between the exosome group and PBS control group under the same treatment time, p<0.05).

**
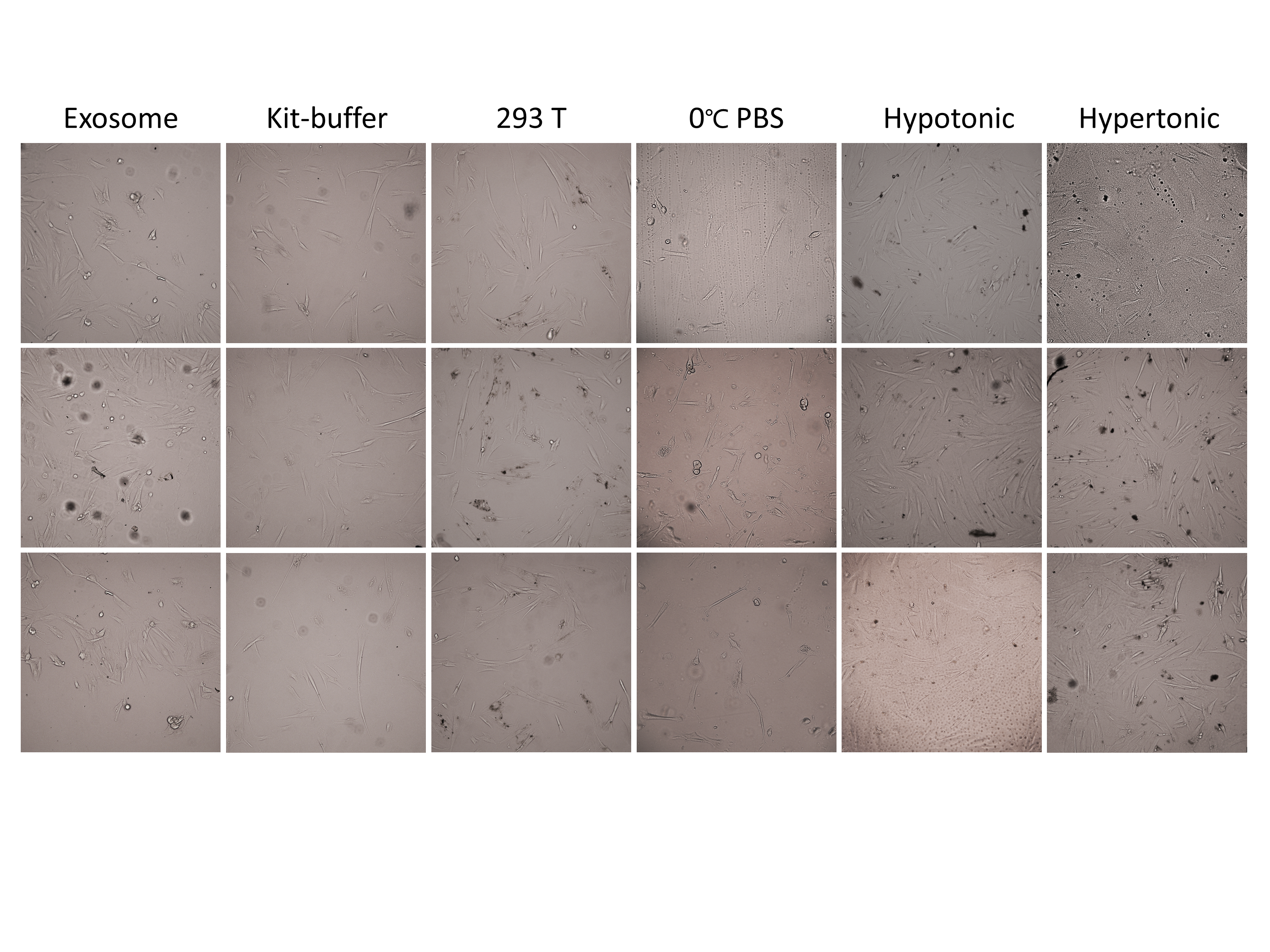
**

**Supplementary Fig. 10.** Changes in cell morphology after removal of mifepristone and addition 100 µL of different solution for 3 h. We found that only the MSC exosomes group showed significant cell roundness. 293 T group and Kit-buffer group was the same exosomes extraction process as the MSC exosomes group. The hypertonic group was 600 mOsm, and the hypotonic group was water. The black spots in the bright area were mifepristone, which was used for drug injury, and there was a small residue washed by PBS.

**
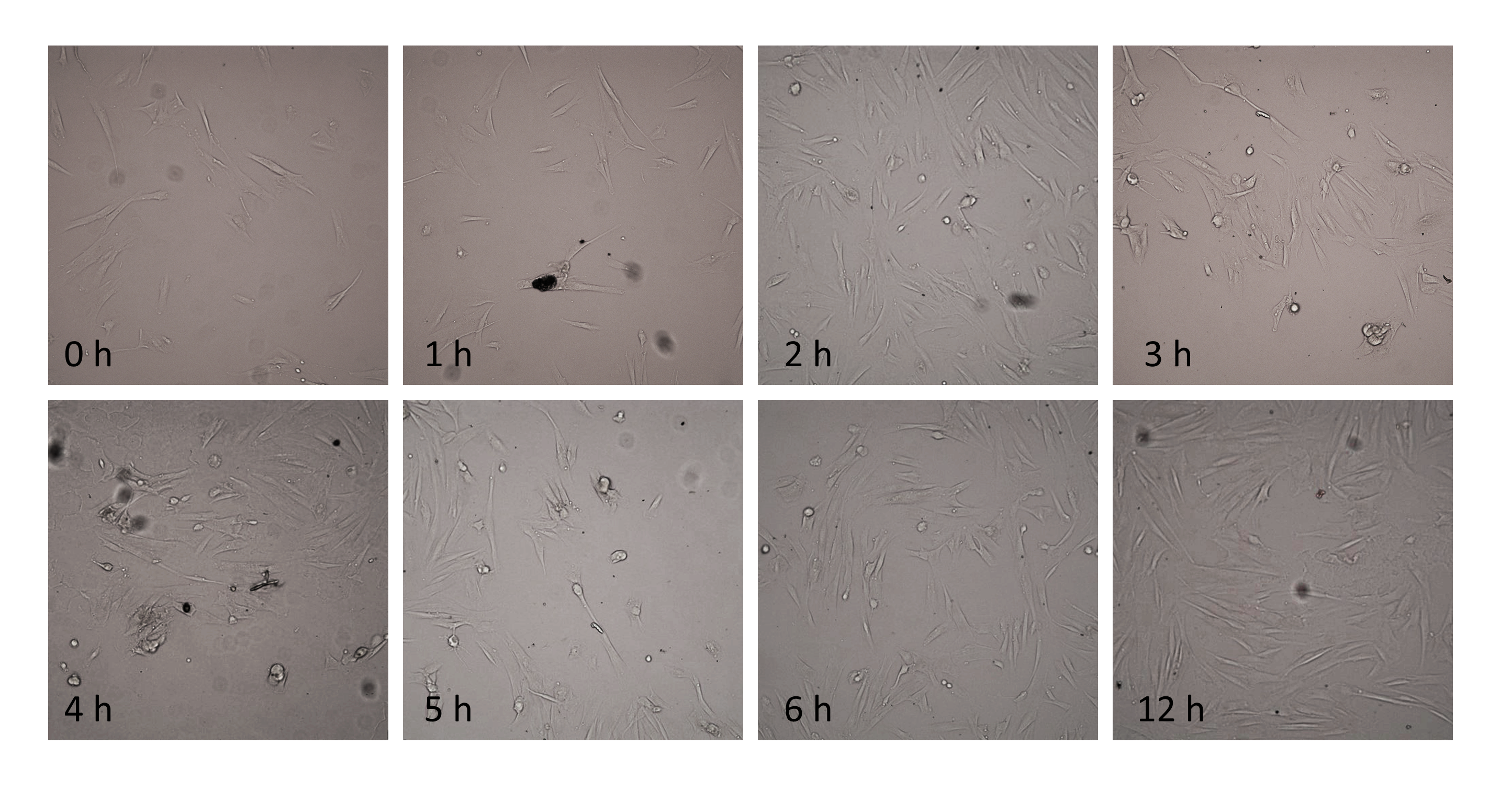
**

**Supplementary Fig. 11.** The morphological changes of ESCs in culture dish after MSC exosomes action.

**
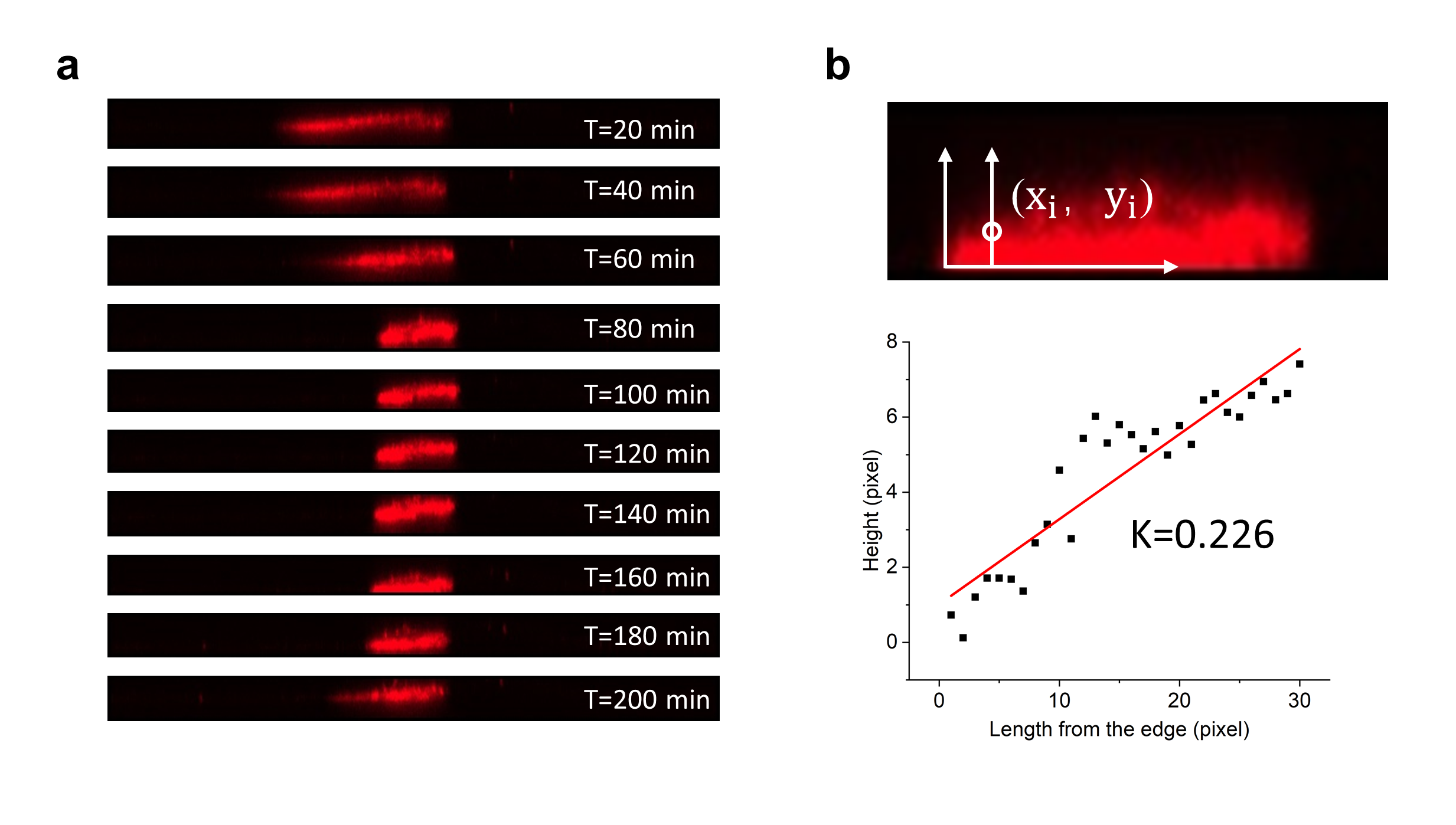
**

**Supplementary Fig.12.a,** Reconstruction results of Z-direction F-actin immunofluorescence staining, the normal vector was perpendicular to the cell principle axis. **b,** Starting from the cell edge, the boundary points on the upper side of the cell staining image can be obtained. By linear fitting, the slope value can reflect the inclination of microfilaments.


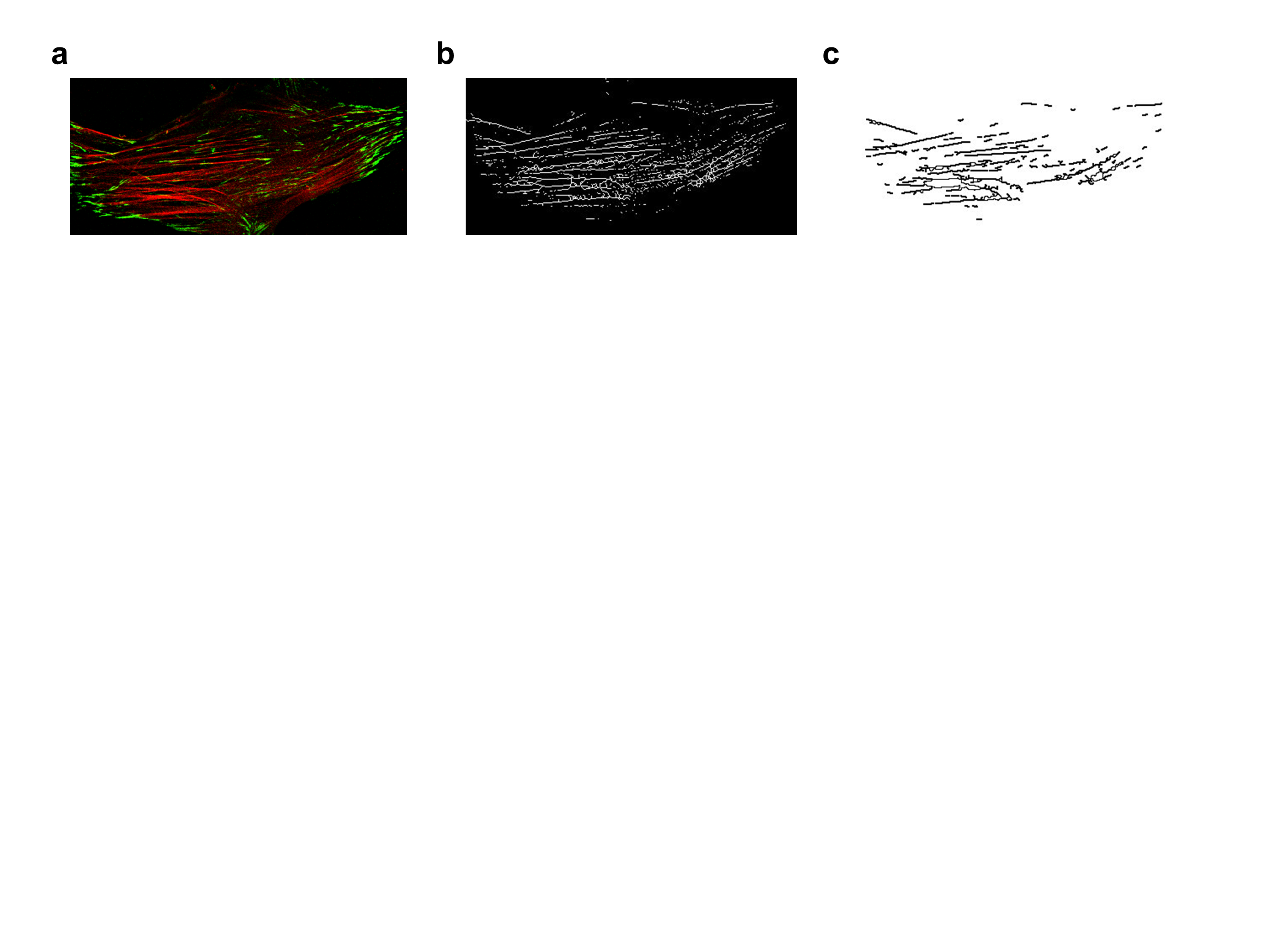


**Supplementary Fig. 13.a,** Z-stack maximum intensity projection of paxillin (green) and F-actin (red) staining within an EndoSC. **b,** Followed by iterative graph matching of skeletonized curvilinear features in close proximity and orientation to extract a map of network fragments of various lengths. **c,** The appropriate length threshold was determined according to the position of the focal adhesion, and the stress fiber network was screened for length and quantity statistics.

**
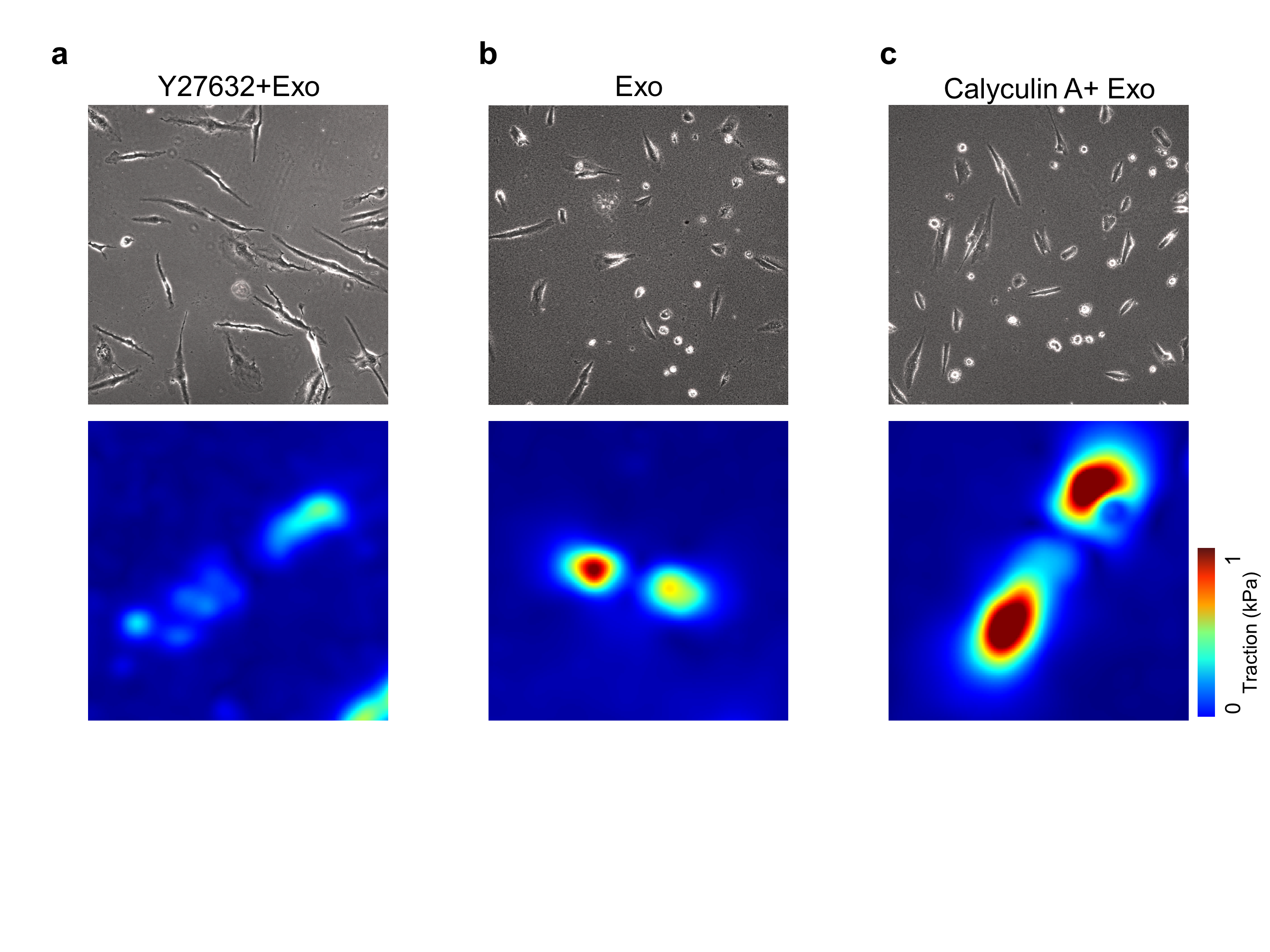
**

**Supplementary Fig. 14.a,** Cell morphology and representative tractive results with the treatment of Y27632 combined with exosomes for 3 h. **b,** With exosomes. **c,** With calyculin A and exosomes.

**
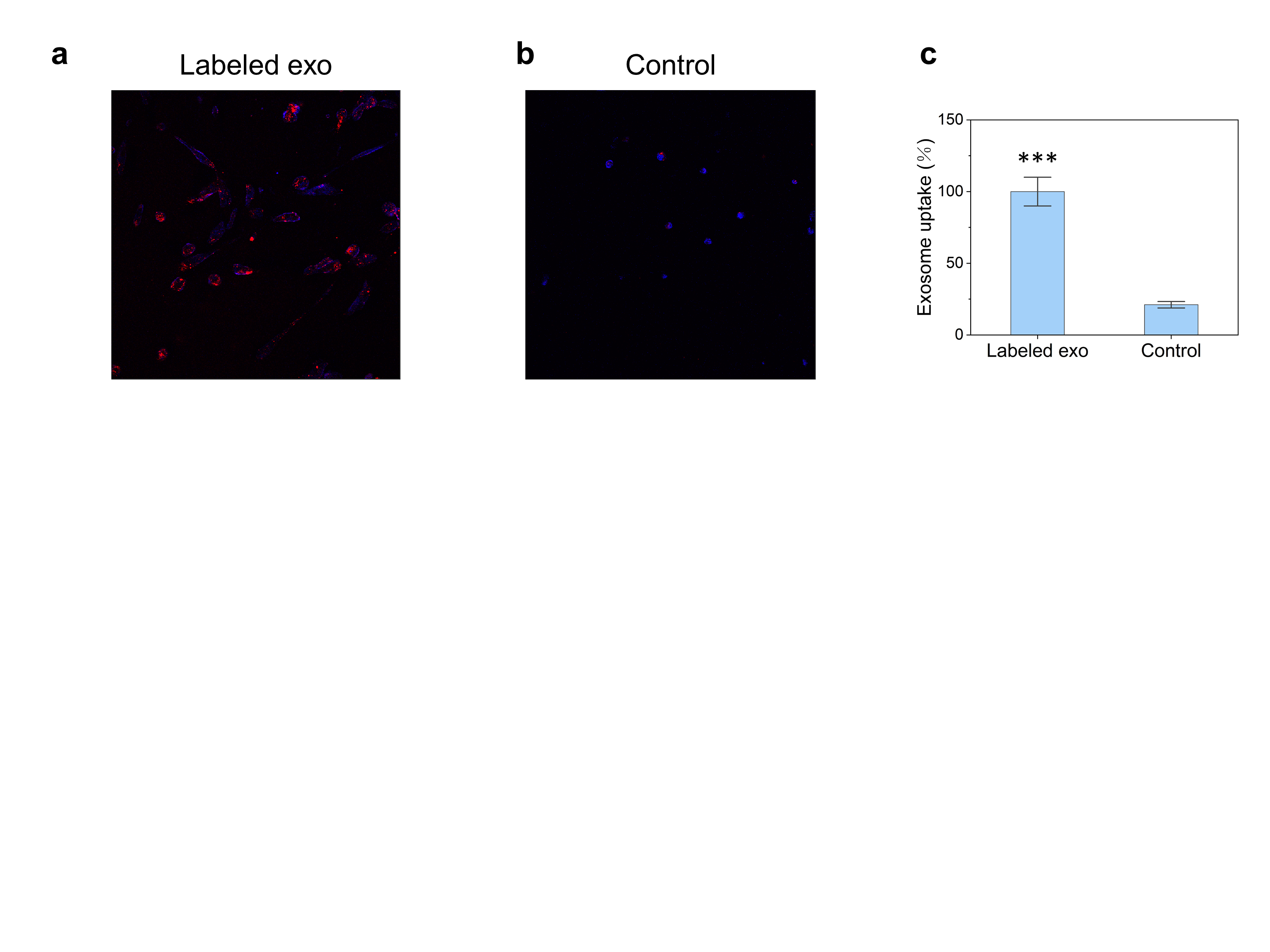
**

**Supplementary Fig. 15.a,** Confocal images of EndoSCs treated with labeled exosomes (red) for 3 h, the fluorescence intensity of exosomes was quantified by mapping the cell region via bright field and nucleus (blue). **b,** In the control group, We used PBS to replace the exosome extract solution, and other experimental procedures remained the same. **c,** The statistical results showed that the staining results were mainly derived from the specific signals of exosomes.

**Supplementary Video 1**

Cell traction force mapping of endometrial stromal cells treated with mifepristone, mifepristone was added at t=8 min. Scale bar, 20 μm.

**Supplementary Video 2**

Cell traction force mapping of injured endometrial stromal cells treated with exosomes, exosomes was added at t=0 min. Scale bar, 20 μm.

**Supplementary Video 3**

Cell traction force mapping of injured endometrial stromal cells treated with exosomes, which showed transient cell rounding, exosomes was added at t=0 min. Scale bar, 20 μm.

**Supplementary Video 4**

Endometrial stromal cells contracted and rounded in the early stages of exosomes treatment on 5.8 kPa PAA gels. Scale bar, 50 μm.

**Supplementary Video 5**

Live cell microfilament staining for endometrial stromal cells in the early stages of exosomes treatment. The distribution of the cytoskeleton changes markedly when the briefly rounded cells (yellow arrows point out the cells) spread out. Video started with the exosome adding 3 h, recorded at 12 frame h^-1^. Scale bar, 20 μm.
